# Whole-body adipose tissue multi-omic analyses in sheep reveal molecular mechanisms underlying local adaptation to extreme environments

**DOI:** 10.1038/s42003-023-04523-9

**Published:** 2023-02-08

**Authors:** Ya-Xi Xu, Bo Wang, Jia-Nan Jing, Rui Ma, Yan-Hui Luo, Xin Li, Ze Yan, Ya-Jing Liu, Lei Gao, Yan-Ling Ren, Meng-Hua Li, Feng-Hua Lv

**Affiliations:** 1 College of Animal Science and Technology, China Agricultural University, Beijing, 100193 China; 2 Institute of Animal Husbandry and Veterinary Medicine, Xinjiang Academy of Agricultural and Reclamation Sciences, Shihezi, China; 3 Shandong Binzhou Academy of Animal Sciences and Veterinary Medicine, Binzhou, China

**Keywords:** Gene regulation, Agricultural genetics

## Abstract

The fat tail of sheep is an important organ that has evolved to adapt to extreme environments. However, the genetic mechanisms underlying the fat tail phenotype remain poorly understood. Here, we characterize transcriptome and lipidome profiles and morphological changes in 250 adipose tissues from two thin-tailed and three fat-tailed sheep populations in summer and winter. We implement whole-genome selective sweep tests to identify genetic variants related to fat-tails. We identify a set of functional genes that show differential expression in the tail fat of fat-tailed and thin-tailed sheep in summer and winter. These genes are significantly enriched in pathways, such as lipid metabolism, extracellular matrix (ECM) remodeling, molecular transport, and inflammatory response. In contrast to thin-tailed sheep, tail fat from fat-tailed sheep show slighter changes in adipocyte size, ECM remodeling, and lipid metabolism, and had less inflammation in response to seasonal changes, indicating improved homeostasis. Whole-genome selective sweep tests identify genes involved in preadipocyte commitment (e.g., *BMP2, PDGFD*) and terminal adipogenic differentiation (e.g., *VEGFA*), which could contribute to enhanced adipocyte hyperplasia. Altogether, we establish a model of regulatory networks regulating adipose homeostasis in sheep tails. These findings improve our understanding of how adipose homeostasis is maintained, in response to extreme environments in animals.

## Abstract (web summary)

Survival in extreme environments and adaptations during seasonal shifts require specialized metabolic programing. A multi-omic analysis of how regulatory networks impact adipose homeostasis in sheep tails provides insight for how these animals survive extreme environments.

## Introduction

To cope with environmental stress such as cold, heat, food scarcity, and high altitude, animals have evolved physiological functions and/or organs to increase energy reserves and reduce energy consumption^1–3^. In fact, adipose tissue is an important and active endocrine organ that animals use to survive in extreme environments^3^. In contrast to those living in mild environments, animals (e.g., bears, camels, and fat-tailed sheep) surviving in extreme environments typically have functionally specialized or extra adipose depots^4–6^. Adipose tissues can change in size dramatically in response to an energy surplus or deficit and are highly plastic and dynamic. This process is referred to as adipose tissue remodeling, which includes adipocyte hyperplasia and hypertrophy, degradation and rebuilding of extracellular matrix (ECM), infiltration of macrophages, and remodeling of blood vessels^7–9^.

Sheep have developed substantial phenotypic variations to adapt to various environments during domestication and subsequent selection. The adipose tissues of fat-tailed sheep tend to have evolved high plasticity and a strong capacity for the storage and mobilization of energy, while humans face the growing epidemic of obesity and associated metabolic syndrome^10,11^. In sheep, fat tail is one of the traits that has evolved to adapt to the steppe and desert conditions^12^. Understanding the underlying energy homeostasis in response to environmental challenges could have applications for adapting to climate change. Previous studies revealed a few candidate genes associated with the fat tail phenotype using multiomics^13–17^. For example, candidate genes *PDGFD*, *BMP2*, *TBXT,* and *HOXB13* were detected by selective sweep or GWAS analysis^15,18–22^, which are involved in preadipocyte differentiation and associated with fat deposition in sheep^5,23^. In addition, many candidate genes, microRNA and lncRNA were identified by transcriptomic and proteomic approaches, such as *PGDFD*, *BMP2*, *IL6*, *ADIPOQ*, *miR-432*, most of which are related to fat deposition and lipid metabolism^24–28^. Those studies revealed plenty of candidate genes and regulatory elements associated with the fat tail phenotype and suggested a complex genetic mechanism for the fat tail phenotype. However, the genetic and molecular mechanisms by which fat-tailed sheep maintain metabolic homeostasis under different nutritional and environmental conditions remain to be elucidated.

Here, we selected 50 ewes (2–3 years old) of Tibetan sheep, Chinese Merino, Small-tailed Han, Wadi, and Altay sheep. Tibetan sheep and Chinese Merino are thin-tailed breeds, of which Tibetan sheep is a typical short and thin-tailed breed and have adapted to the Qinghai–Tibetan Plateau environments (cold and hypoxic)^29^. Chinese Merino, the well-known sheep for fine wool production in China, is a typically long thin-tailed breed and has adapted themselves to local climate of Xinjiang of China^30,31^. Small-tailed Han, Wadi, and Altay sheep are native fat-tailed breeds in China with tail configurations. Small-tailed Han sheep is a short fat-tailed breed, inhabiting various environments due to their high fertility and good adaptation in China^32^. Wadi and Altay sheep are local breeds with long fat-tail and fat-rump, respectively. Also, Wadi sheep is a prolific local breed limited in Shandong Province of China, while Altay sheep is well adapted to the dry, cold mountain basins climate in Xinjiang of China^33,34^. In this study, we characterized the transcriptomic and lipidomic profiles of adipose tissues located in the five depots of fat- and thin-tailed sheep populations in summer and winter. The five adipose tissues covered two main adipose types of adult mammals, subcutaneous and visceral adipose tissues classified by their anatomical locations^35^ and tail adipose tissue, and played important biological functions in sheep, such as metabolic dysfunction, lipolysis, and fat deposition^36–38^. Combining these data with whole-genome sequence data, we aimed to reveal the molecular regulatory mechanisms underlying the difference in fat deposition capacities between the tails of different sheep populations in different seasons. In addition, this study improves our understanding of genetic adaptations to extreme environments in this and other animal species.

## Results

### RNA-Seq data

**Fig. 1:**
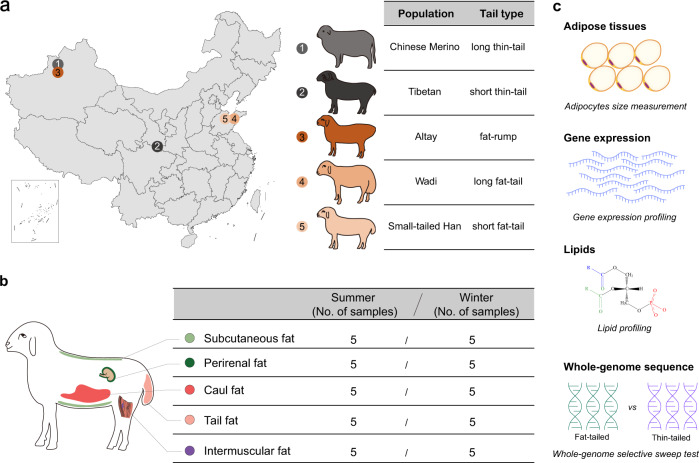
Schematic overview of the study design. **a** Sample population information; **b** Sample tissue information; **c** Illustration of the data types used in the study. Histological analysis was used to measure adipocyte size; gene expression and lipid profiles were quantified by transcriptomic and lipidomic analyses; and whole-genome sequence data was used to identify candidate genome regions and genes under selection.

Across all 250 adipose tissue samples, a total of 1780 Gb of clean reads were retained with an average of 23.7 million reads (14.6–43.5 million reads) per sample after quality control (Fig. 1a, b and Supplementary Data 1, 2). Of the total clean reads, 83.42–97.42% of reads were mapped to the sheep reference *Oar_Rambouillet v1.0* and 72.72–90.21% showed unique hits among tissues (Supplementary Data 2). We identified a total of 27,685 protein-coding genes. The PCA (Principal Component Analysis) plot of the transcriptome data showed unique clusters of adipose tissues of Tibetan sheep in winter, but no clear differentiation between tissues of different depot origins or seasonal differences between the sample tissues. (Supplementary Fig. 1).

### Differential gene expression between summer and winter samples

**Fig. 2:**
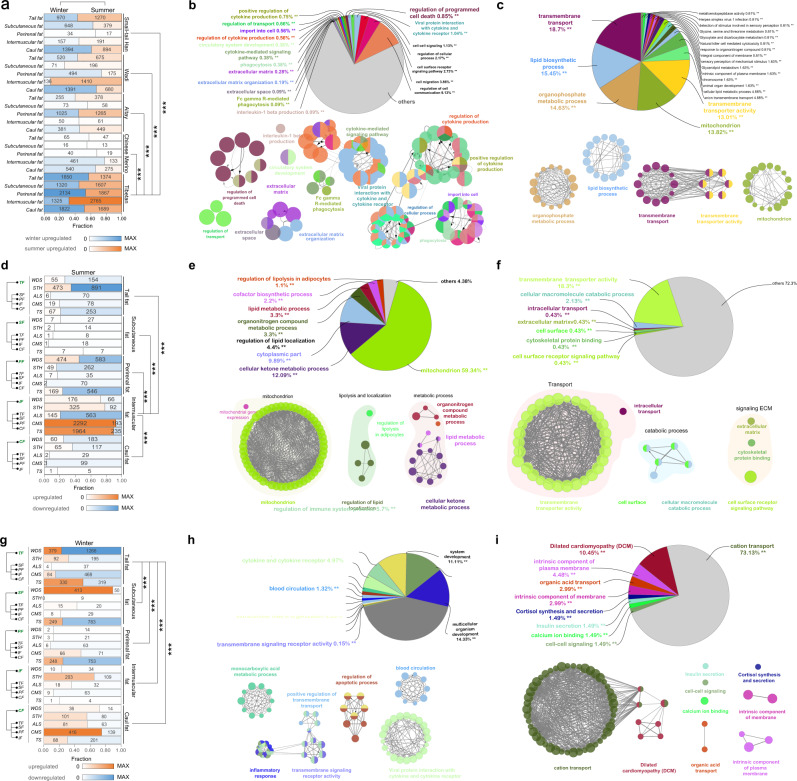
Gene expression patterns and functions affected by the changing seasons and specific tissues. **a** Bar chart of the numbers of DEGs found to be upregulated or downregulated in each type of adipose tissue from five breeds between summer and winter samples. **b** Pie charts of GO terms and KEGG pathways of total upregulated genes identified in five adipose tissues from Tibetan sheep in winter. **c** Pie charts of GO terms and KEGG pathways of total upregulated genes identified in five adipose tissues from Tibetan sheep in summer. **d** Bar chart of the numbers of upregulated or downregulated genes in each type of adipose tissue from five breeds compared to the other four tissue samples in summer. **e** Pie charts of GO terms and KEGG pathways of total downregulated genes identified in intermuscular fat (IMF) from five breeds in summer. **f** Pie charts of GO terms and KEGG pathways of total upregulated genes identified in intermuscular fat (IMF) from five breeds in summer. **g** Bar chart of the numbers of upregulated or downregulated genes in each type of adipose tissue from five breeds compared to the other four tissue samples in winter. **h** Pie charts of GO terms and KEGG pathways of total downregulated genes identified in tail fat (TAF) in winter. **i** Pie charts of GO terms and KEGG pathways of total upregulated genes identified in tail fat (TAF) in winter. ***P value < 0.001; **P value < 0.01. These plots are based on results in Supplementary Data 3–5, 10–14.

We examined the difference in gene expression profiles of adipose tissues during seasonal changes. Across all the comparisons among the five fat depots in the five populations, the range of DEGs identified between summer and winter ranged from 13 (for subcutaneous fat of Chinese Merino) and 2756 (for intermuscular fat of Tibetan sheep) (Fig. 2a and Supplementary Data 3). Of note, the five tissues from Tibetan sheep exhibited substantially seasonal differences in gene expression (Fig. 2a). The significantly upregulated genes in winter were mainly enriched in extracellular matrix (e.g., *MMP14*, *PTX3*, and *FBLN5*), molecular transport (e.g., *CAPN3*, *TRPM2*, and *STON2*), cytokines (e.g., *WNT5A*, *CCR5*, and *CXCL10*), phagocytosis (e.g., *COLEC12*, *FCGR1A*, and *VAV1*), and circulatory system (e.g., *C3AR1*, *WNT5A*, and *STAT1*) (Fig. 2b and Supplementary Data 4), while the significantly upregulated genes in summer were mainly enriched in molecular transport (e.g., *SLC24A3*, *SFXN5*, and *ANKH*), lipid metabolism and biosynthetic process (e.g., *DGAT2*, *SCD*, and *LEP*), and mitochondrion (e.g., *ACSM3*, *PINK1*, and *SLC25A33*) (Fig. 2c and Supplementary Data 5). This observation is supportive of the concept that differentially expressed genes (DEGs) between seasons in Tibetan sheep were associated with adipose tissue remodeling. Substantial variations were detected in the gene expression patterns of different populations. Only 329 and 181 upregulated genes across the five tissues were discovered in Tibetan sheep in summer and winter, respectively. In addition, only a small number of common genes among the five tissues were detected in the remaining four populations (Supplementary Fig. 2). However, enrichment analyses of DEGs showed that the DEGs were functionally similar among different populations and were mainly related to catabolic and metabolic processes (e.g., *SCD, ELOVL6*, and *ADIPOQ*), blood circulation (e.g., *WNT5A*, *BMP4*, and *CXCL10*), homeostasis (e.g., *CAPN3*, *C3AR1*, and *ELOVL6*), inflammatory response (e.g., *CCR5*, *TNFAIP6*, and *IL17RB*), thermogenesis (e.g., *UCP1*, *DECR1*, and *UCP2*), mitochondrion (e.g., *TMBIM6*, *UCP1*, and *CIDEA*), ribosome (e.g., *RPL37*, *RPS27*, and *RPS3*), and molecular transport (e.g., *CAPN3*, *SFXN5*, and *PTX3*) (Supplementary Figs. 3–6 and Supplementary Data 6, 7). Moreover, and as expected, functional genes which showed significantly differential expressions in adipose tissues of tail fat of fat-tailed and thin-tailed sheep populations between summer and winter were significantly enriched in gene ontology and pathways involved in lipid metabolism (e.g., *DGAT1* and *PNPLA2*), ECM remodeling (e.g., *FBN1* and *WNT5A*), cellular molecular transport (e.g., *ADIPOQ* and *IGF1*), inflammatory response (e.g., *TNF* and *IFNG*), cytokines (e.g., *TGFB3* and *IL10*), and blood circulation (e.g., *COL1A2* and *PPARG*) (Supplementary Data 8, 9). This observation is consistent with adipose tissue homeostasis.

### Tissue-specific expression patterns in different seasons

We evaluated depot-specific gene expression by comparing one fat depot to the other four. Across all the comparisons among the five adipose tissues in the five populations, the range of DEGs identified among tissues ranged from 1 (for subcutaneous fat of Altay and Chinese Merino, and caul fat of Tibetan sheep) and 2292 (for intermuscular fat of Chinese Merino) in summer (Fig. 2d, Supplementary Fig. 7 and Supplementary Data 10). Of the 50 pairwise comparisons, two comparisons of the intermuscular adipose tissues of the two thin-tailed sheep populations detected more DEGs (*n* = 2199–2485) than the others (*n* = 6–1364). These DEGs had important functions in cellular molecular transport (e.g., *MLC1*, *TRIM15*, and *SLC7A10*), ECM remodeling (e.g., *COCH*, *WNT3*, and *LAMA1*), catabolic processes (e.g., *MT3*, *USP43*, *NRG1*, and *TIMP1*) mitochondria (*ACSM3*, *NDUFA7*, and *MRPL28*), metabolic processes (*ISL2*, *PAX2*, *HOXB5*, and *HOXB3*), lipolysis (*PLIN1*, *ADRB1*, and *LIPE*), and lipid localization (*SPP1, MSR1*, and *ADIPOQ*) according to the enrichment analysis (Fig. 2e, f and Supplementary Data 11, 12). For the remaining 48 comparisons, relatively few tissue-specific DEGs were identified (Fig. 2d), but enrichment annotation of the DEGs showed functions similar to those described above, including inflammatory response (e.g., *CCL22*, *ICOS*, and *CCR7*), cytokines (e.g., *CXCL14*, *CCL22*, and *IL6*), ECM remodeling (e.g., *MMP27*, *PTX3*, and *WNT2*), cellular molecular transport (e.g., *SLC5A7*, *ABCC12*, and *CHRNA2*), biosynthetic process (e.g., *PTX3*, *ELOVL3*, and *SLC1A1*), and metabolic process (e.g., *UCP1*, *UMOD*, and *CCR7*) (Supplementary Figs. 8–12 and Supplementary Data 8–12).

For the adipose tissues sampled in winter across all 25 comparisons, fewer tissue-specific DEGs were identified than in summer (Fig. 2g and Supplementary Data 10). Among the tissues, the tail fat-specific expression profiles were significantly (*p*_adj_ < 0.05) different from the others, with more tail fat-specific expressed genes. The highly expressed genes were significantly (*p*_adj_ < 0.05) enriched in GO terms and KEGG pathways such as cation transport (e.g., *TNNI3, SLC5A1*, and *STC2*), cell signaling (e.g., *DLK1, WNT10A*, and *GATA5*), organic acid transport (e.g., *SLC6A15, LRP2*, and *ABCC2*), and inflammatory response (e.g., *PAX1, ISL1*, and *CCL19*) (Fig. 2h, i and Supplementary Data 13, 14). In tissues from the other fat depots, the depot-specific DEGs were significantly (*p*_adj_ < 0.05) enriched in several of the same GO terms and KEGG pathways described above (Supplementary Figs. 12–15 and Supplementary Data 15–18).

### Global lipidomic profiles of sheep adipose tissues

**Fig. 3:**
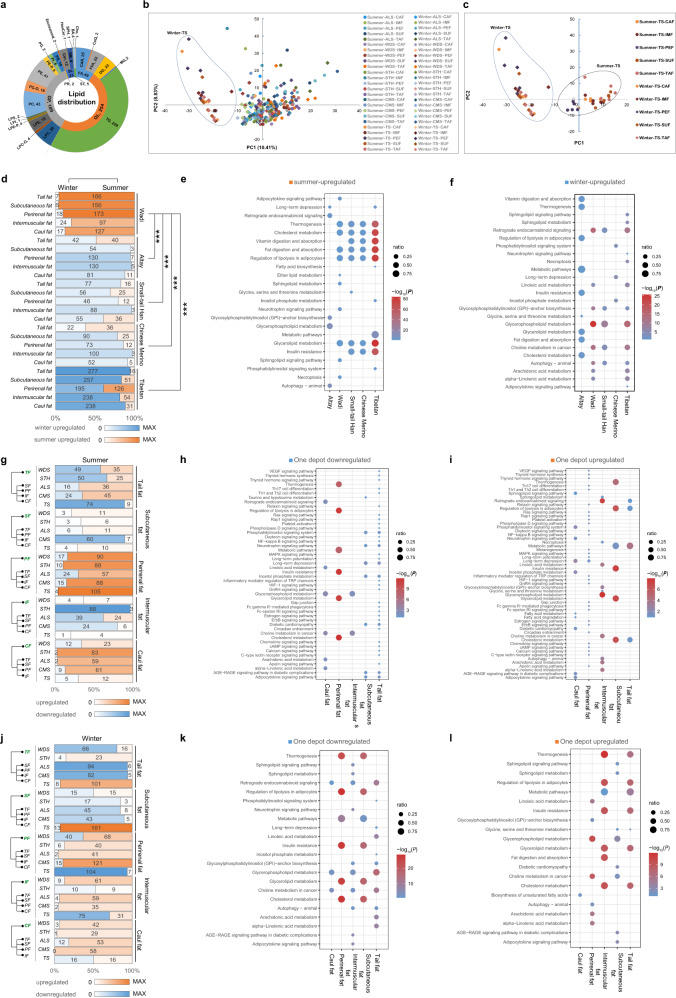
Lipidomic analysis reveals differentially expressed lipids (DELs) and their functional pathways in adipose tissues from different seasons and tissue locations in 5 sheep populations. DELs were identified by *P*_adj_ value < 0.05 and |log_2_FC| ≥ 1; ***P < 0.001. **a** Lipids composition**. b** Principal component analysis (PCA) plot showing the variation among 50 adipose tissue groups using lipidomic data. **c** PCA plot showing the variation among 10 adipose tissue groups of Tibetan sheep using lipidomic data. **d** DELs of comparisons between adipose tissues in summer and winter. **e** GO and KEGG pathways enriched with lipids upregulated in summer. **f** GO and KEGG pathways enriched with lipids upregulated in winter. **g** DELs of comparisons between one adipose tissue and the other four in summer. **h** GO and KEGG pathways enriched with tissue-downregulated lipids in summer. **i** GO and KEGG pathways enriched with tissue-upregulated lipids in summer. **j** DELs of comparisons between one adipose tissue and the other four in winter. **k** GO and KEGG pathways enriched with tissue-downregulated lipids in winter. **l** GO and KEGG pathways enriched with tissue-upregulated lipids in winter. These plots are based on results in Supplementary Data 19–28.

Lipidomic profiles of 250 adipose tissue samples identified a total of 522 lipids of six classes, including glycerolipids (GL), glycerophospholipids (GP), fatty acyls (FA), sphingolipids (SL), Sterol lipids (ST), and prenol lipids (PR) (Fig. 3a and Supplementary Data 19). PCA of the lipids revealed two distinct groups, which is consistent with the pattern identified in the PCA plot of the RNA-Seq data (Fig. 3b, c and Supplementary Data 20).

To reveal the seasonal variations in lipid profiles of adipose tissues, the supervised partial least squares-discriminant analysis (PLS-DA) multivariate method was used to detect significant differences in lipid abundance between the summer and winter samples. For the 25 total comparisons of a particular adipose depot within each population, the range of significant differences in lipid abundance (VIP ≥ 1 and absolute log_2_FC (fold change) ≥1) identified between summer and winter ranged from 3 (for subcutaneous fat of Altay sheep) and 238 (for intermuscular fat and caul fat of Tibetan sheep) (Fig. 3d and Supplementary Data 21). Although the relative abundance of lipids showed differences among tissues and populations (Fig. 3d), the significantly upregulated lipid metabolites in summer were related to several metabolic pathways such as thermogenesis, cholesterol metabolism, fat digestion and absorption, and glycerolipid metabolism (Fig. 3e and Supplementary Data 22). The significantly upregulated lipid metabolites in winter were significantly enriched in metabolic pathways such as the regulation of lipolysis in adipocytes, vitamin digestion and absorption, metabolic pathways, and insulin resistance (Fig. 3f and Supplementary Data 23). Notably, we observed different functions of the differential metabolites in Altay sheep (Fig. 3e, f). These functions of significantly upregulated lipids were consistent with those of significantly upregulated DEGs from transcriptome analyses of winter and summer samples.

In the lipidomic analyses, we detected depot-specific lipid abundance in different seasons by comparing one adipose tissue to the other four. In the summer samples, across all the comparisons among the five adipose tissues in the five populations, the range of significant differences in lipid abundance (VIP ≥ 1 and absolute log_2_FC (fold change) ≥1) identified among populations ranged from 1 (for intermuscular fat of Tibetan sheep) and 105 (for perirenal fat of Tibetan sheep) (Fig. 3g and Supplementary Data 24). The upregulated and downregulated lipid metabolites were related to thermogenesis, regulation of lipolysis in adipocytes, metabolic pathways, and glycerolipid metabolism (Fig. 3h, i and Supplementary Data 25, 26), which was consistent with the significantly enriched pathways of the upregulated and downregulated DEGs in the transcriptome analyses in the summer samples (Fig. 2e, f).

In the winter samples, across all the comparisons among the five adipose tissues in the five populations, the range of significant differences in lipid abundance (VIP ≥ 1 and absolute log_2_FC (fold change) ≥1) identified among populations ranged from 0 (for caul fat of Chinese Merino) and 161 (for subcutaneous fat of Tibetan sheep) (Fig. 3j and Supplementary Data 24). The upregulated and downregulated lipid metabolites (VIP ≥ 1 and absolute Log_2_FC (fold change) ≥ 1) were significantly enriched in pathways such as thermogenesis, metabolic pathways, regulation of lipolysis in adipocytes, insulin resistance, and the adipocytokine signaling pathway (Fig. 3k, l and Supplementary Data 27, 28), which was consistent with the functions of upregulated and downregulated genes observed in the transcriptomic analysis in the winter samples (Fig. 2h, i).

### Seasonal changes in adipocyte size

**Fig. 4:**
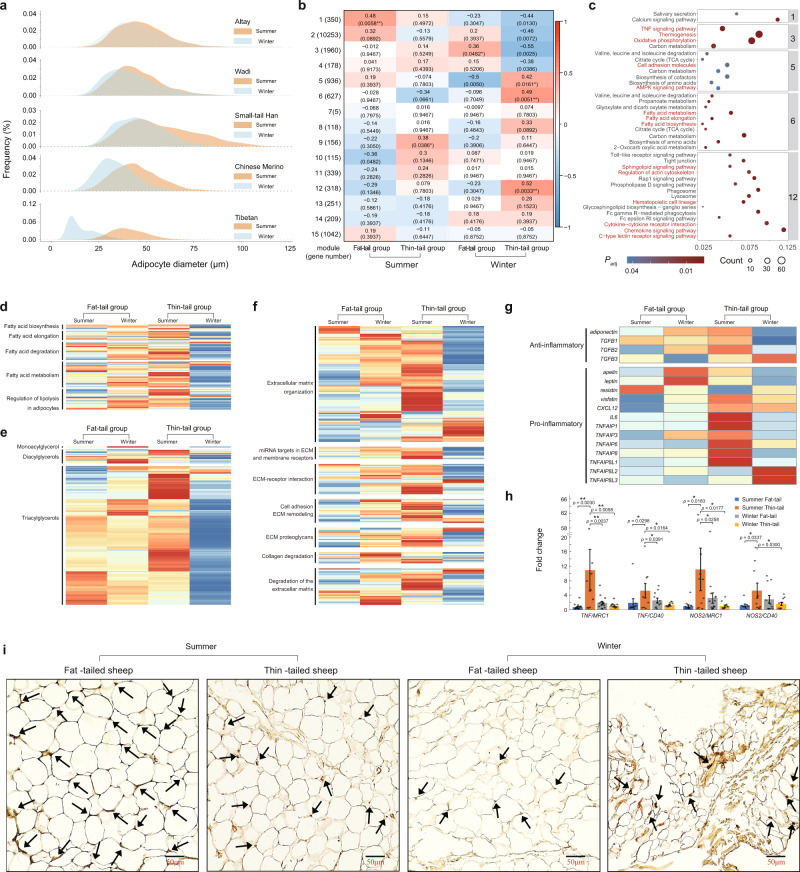
Variations in cell size, metabolism, ECM, and inflammatory reactions among tail adipose tissues with different fat deposition capacities in different seasons. **a** Adipocyte size distribution of tail fat from Altay, Wadi, Small-tailed Han, Chinese Merino, and Tibetan sheep in summer and winter. **b** Correlation between the identified WGCNA modules and the four adipose tissue groups (summer-fat tail, summer-thin tail, winter-fat tail, and winter-thin tail). Modules associated with the traits with correlation coefficient > 0.3 and *P*_adj_ value < 0.05 were considered to be significant at the levels of *P_adj_ value < 0.05 or **P_adj_ value < 0.01. Red and blue colors s indicate significant and nonsignificant correlations with gene expression, respectively. **c** Enrichment of the modules significantly associated with four adipose tissue groups. **d** Expression heatmap of genes in fatty acid metabolism related pathways among the four adipose tissue groups. **e** Heatmap of glycerol composition among the four adipose tissue groups. **f** Expression heatmap of genes in extracellular matrix (ECM) related pathways among the four adipose tissue groups. **g** Expression heatmap of anti- and pro-inflammatory genes among the four adipose tissue groups. **h** Changes in M1/M2 cytokine gene expression (RT‒qPCR) among the four adipose tissue groups. **P value < 0.05; **P value < 0.01; bars represent the standard error of the mean. **i** F4/80 staining in adipose tissues from the four groups of season-tail combinations. These plots (**a**–**c**) are based on results in Supplementary Data 29–32, and plot h is based on results in Supplementary Data 33.

Adipocyte size was quantified to evaluate the physiological states of adipocytes in response to seasonal changes. We observed larger tail adipocyte sizes in summer than in winter across all five populations (Fig. 4a and Supplementary Data 29). Interestingly, seasonal differences in the size of tail adipocytes were much greater in the thin-tailed sheep populations than in the fat-tailed populations (Fig. 4a), indicating well-maintained adipose tissue homeostasis in fat-tailed sheep. In addition, we observed that thin-tailed Tibetan sheep had smaller adipocyte sizes in caul fat (Supplementary Fig. 16a), perirenal fat (Supplementary Fig. 16b), and subcutaneous fat than observed in the respective adipose tissues of all the other populations in winter (Supplementary Fig. 16d). Interestingly, adipocytes from intermuscular fat were larger in winter than in summer in all five populations (Supplementary Fig. 16c). The adipocyte size in caul fat (Supplementary Fig. 16a), perirenal fat (Supplementary Fig. 16b), and subcutaneous fat (Supplementary Fig. 16d) did not show any consistent pattern of changes in the five populations from summer to winter.

### Weighted gene co-expression network analysis

We divided the tail adipose tissues into four groups according to season (summer versus winter)-tail (fat-tailed versus thin-tailed) combinations. We then applied weighted gene co-expression network analysis (WGCNA) to identify the four group-specific gene sets associated with fat deposition regulation. We identified 15 distinct modules containing 5–10,253 genes per module. The module eigengene (ME)-to-group correlations revealed 1, 1, 1, and 3 modules significantly correlated with summer-fat-tailed, summer-thin-tailed, winter-fat-tailed, and winter-thin-tailed, respectively (Fig. 4b and Supplementary Data 30). GO and KEGG enrichment analyses implied that the functions of these modules were associated with metabolism (e.g., thermogenesis and fatty acid metabolism), extracellular matrix (ECM) components (e.g., cytokine–cytokine receptor interaction), and inflammatory reactions (e.g., Fc gamma R - mediated phagocytosis) (Fig. 4c and Supplementary Data 31, 32).

In the thin-tailed populations, genes involved in fatty acid metabolism (e.g., biosynthesis, elongation, degradation) and lipolysis were greatly upregulated in summer (Fig. 4d), which indicated that the tail fat of thin-tailed sheep had higher lipid metabolic activity in summer than in winter. Compared to thin-tailed sheep, the fat-tailed populations had relatively similar expression levels of genes related to lipid metabolism in summer and winter (Fig. 4d). The composition of monoacylglycerols, diacylglycerols, and triacylglycerols in the tail fat of thin-tailed sheep was high in summer but low in winter (Fig. 4e). Meanwhile, the thin-tailed populations had high expression of genes involved in ECM remodeling (e.g., *COL11A2, MMP14*, and *ADAM8*) (Fig. 4f), which was consistent with the enlarged adipocytes observed in summer. In addition, pro-inflammatory genes (e.g., *TNFα*) were highly expressed in the tail fat of thin-tailed sheep in summer (Fig. 4g). Compared to that of the thin-tailed populations, the tail fat of fat-tailed sheep contained more macrophages, particularly in summer (Fig. 4i). However, quantitative RT‒qPCR revealed a higher ratio of inflammatory M1 macrophages/anti-inflammatory M2 macrophages in the thin-tailed populations than that in the fat-tailed populations (Fig. 4h and Supplementary Data 33). In comparison to fat-tailed sheep, the massive expansion of adipocytes in the tail fat of thin-tailed sheep in summer was largely related to inflammation and homeostatic imbalance.

Next, we compared the gene expression in tail adipose tissue among the four season-tail combinations for ECM-related pathways, including extracellular matrix organization, miRNA targets in ECM and membrane receptors, ECM-receptor interaction, cell adhesion ECM remodeling, ECM proteoglycans, collagen degradation, and degradation of the extracellular matrix (Fig. 4f). In most cases, the expression of genes in these ECM-related pathways showed substantially seasonal differences in the adipose tissues of thin-tailed sheep, while the seasonal variations of gene expressions in adipose tissues of fat-tailed sheep were much less. These differences suggested that the tail adipose tissues of fat-tailed sheep have a much stronger ability to maintain homeostasis of lipid metabolism and ECM regulation than do those of thin-tailed sheep.

To validate the accuracy of the RNA-Seq data, the expression patterns of 11 genes in 50 tail adipose tissues (fat and thin tail adipose tissues) sampled from winter and summer related to ECM remodeling, lipid droplet dynamic, and inflammation, were quantified using RT-qPCR. The expression level of the 11 genes showed almost the same pattern with RNA-seq data (Supplementary Fig. 17a and Supplementary Data 34), which indicated a close correlation between the results of RT-qPCR and RNA-seq data. Among the 11 genes, *MMP15*, *ADAMTS1*, *STAT3*, *PPARG*, and *LEP* showed significantly higher expression in summer thin-tail adipose tissues than in winter thin-tail adipose tissues, which validated the low-level homeostasis in thin-tail adipose tissues during the seasonal change (Supplementary Fig. 17b and Supplementary Data 34).

### Expression of anti- and pro-inflammatory genes

In addition, we observed the expression of anti- and pro-inflammatory genes in the four adipose tissue groups (Fig. 4g). During the seasonal change from summer to winter, the expression of anti-inflammatory genes mostly increased in the fat-tailed sheep but decreased in the thin-tailed sheep. The expression of pro-inflammatory genes in fat-tailed sheep showed a small difference between summer and winter, while most pro-inflammatory genes in thin-tailed sheep showed much higher expression in summer samples than in winter samples. In addition, the expression of inflammatory genes in the adipose tissues of fat-tailed sheep showed much stronger homeostasis during the seasonal change than that in the adipose tissues of thin-tailed sheep (Fig. 4g).

To further characterize the macrophages present in the four groups of tail-season combinations (e.g., fat-tailed sheep in summer, thin-tailed sheep in summer, fat-tailed sheep in winter, and thin-tailed sheep in winter), the gene expression of classically activated macrophage (M1) versus alternatively activated macrophage (M2) markers was assessed by RT‒qPCR (Fig. 4h, Supplementary Table 1 and Supplementary Data 33). M1/M2 ratios determined using *TNF*/*MRC1*, *TNF*/*CD40*, *NOS2*/*MRC1*, and *NOS2*/*CD40* showed the same tendency among the four groups (Fig. 4h). The M1/M2 expression ratio was the highest in the thin-tailed sheep in summer, while it decreased considerably in winter (Fig. 4h). However, the expression ratio of M1/M2 markers in fat-tailed sheep was the lowest in summer, while it increased greatly in winter.

Next, adipose tissues from the tails were immunostained with F4/80 antibody (1:500 dilution), a macrophage surface maker (Fig. 4i). Compared with adipose tissues from the tails of thin-tailed sheep, adipose tissues from the tails of fat-tailed sheep in summer showed much higher macrophage densities, which decreased considerably in winter. In winter, higher macrophage densities were observed in the adipose tissues of thin-tailed sheep, which might be a consequence of the reduced volume of the fat pads.

### Whole-genome selective signals associated with the fat-tail phenotype

**Fig. 5:**
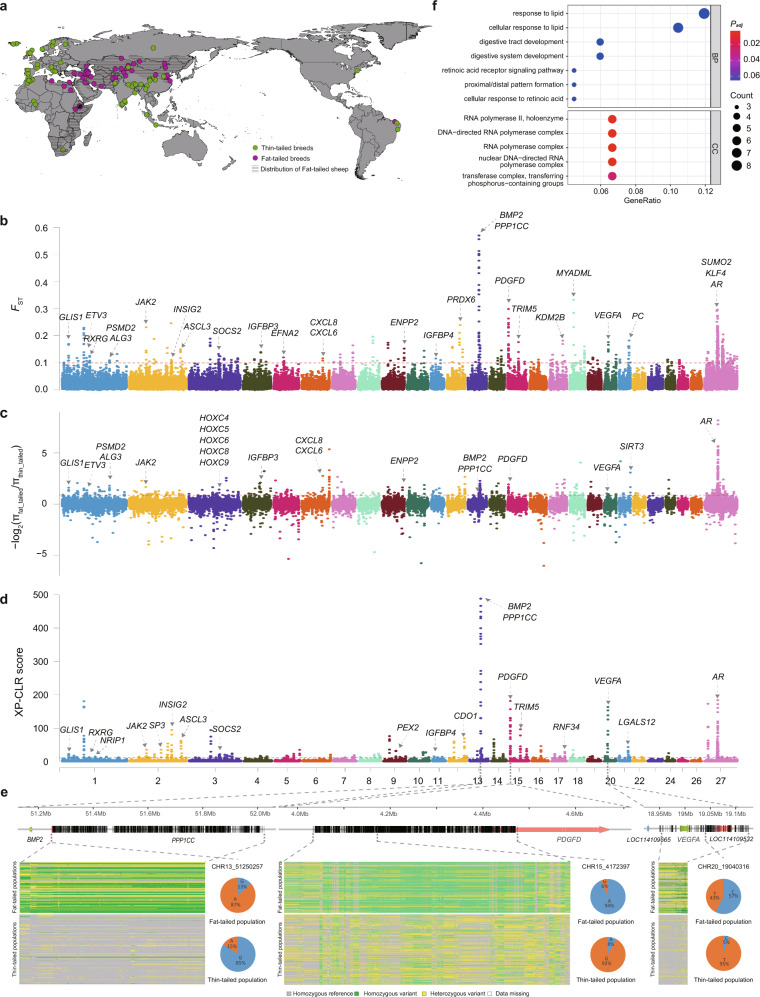
Genome-wide selective signals in fat-tailed sheep. **a** Sampling locations of the 221 fat-tailed sheep and 304 thin-tailed sheep, and geographic distribution ranges of fat-tailed sheep. **b** Whole-genome selective signals between fat-tailed sheep and thin-tailed sheep based on the pairwise *F*_ST_ selection test. **c** Whole-genome candidate selective regions between fat-tailed sheep and thin-tailed sheep by the log_2_(π_ratio_) selection test. **d** Genome-wide selective signals between fat-tailed sheep and thin-tailed sheep by the cross-population composite likelihood ratio (XP-CLR) test. The horizontal red dashed line corresponds to the genome-wide significance threshold (top 5‰: *F*_ST_ = 0.0989, log_2_(π_ratio_) = 0.8686, and XP-CLR = 11.0528). **e** Schematic diagram of the gene structure of the *BMP2*, *PPP1CC*, *PGDFD*, and *VEGF* genes (top); the genotype patterns of the *PPP1CC*, *PGDFD*, and *VEGF* genes among fat-tailed and thin-tailed populations (left); pie charts (right) represent the allele frequencies at the most associated loci in the selective regions among fat-tailed and thin-tailed populations; derived alleles are indicated in blue and the reference alleles are marked in red. **f** GO terms enriched based on genes identified by at least two of the three methods [XP-CLR, log_2_(π_ratio_), and *F*_ST_]. These plots are based on results in Supplementary Data 35–45.

After quality control, we obtained a total of 26,493,595 SNPs in 525 genomes of sheep from around the world (Fig. 5a and Supplementary Data 35). We first examined population differentiation using principal component analysis (PCA), phylogenetic tree, and sNMF approaches. The principal component analysis shows a clear separation between fat-tailed and thin-tailed sheep (Supplementary Fig. 18), a pattern further supported by the phylogenetic tree (Supplementary Fig. 19) and sNMF analysis (Supplementary Fig. 20). The results revealed a common genetic origin for fat-tailed sheep sampled in this study (Small-tailed Han, Wadi, and Altay sheep) (Supplementary Figs. 18–20). Nevertheless, fat-tailed sheep collected in this study (Tibetan sheep and Chinese Merino) showed a certain level of degree of genetic differentiation (Supplementary Figs. 18–20). Genome-wide pairwise *F*_ST_, *π*_ratio_, XP-CLR score values, and XP-EHH sore values between fat-tailed and thin-tailed populations were calculated (Fig. 5b–d and Supplementary Fig. 21). We identified 139, 217, 161, and 123 candidate genomic regions based on the top 5‰ of *F*_ST_ (0.099), log_2_(*π*_ratio_) (0.8686), XP-CLR scores (10.78), and XP-EHH scores (0.4559), respectively (Supplementary Data 36–39). Using the first 3 methods, 98 selective sweep regions were detected with at least two methods, which represent 0.41% (11.55 Mb) of the genome, covering 404 genes (Supplementary Data 40–43). The selective regions detected by XP-EHH showed a similar pattern to those found by *F*_ST_, XP-CLR, and log_2_(*π*_ratio_). Overall, 40, 67, and 43 overlapping regions were detected by XP-EHH and *F*_ST_, XP-CLR, and log_2_(*π*_ratio_), respectively (Supplementary Data 44–45).

**Fig. 6:**
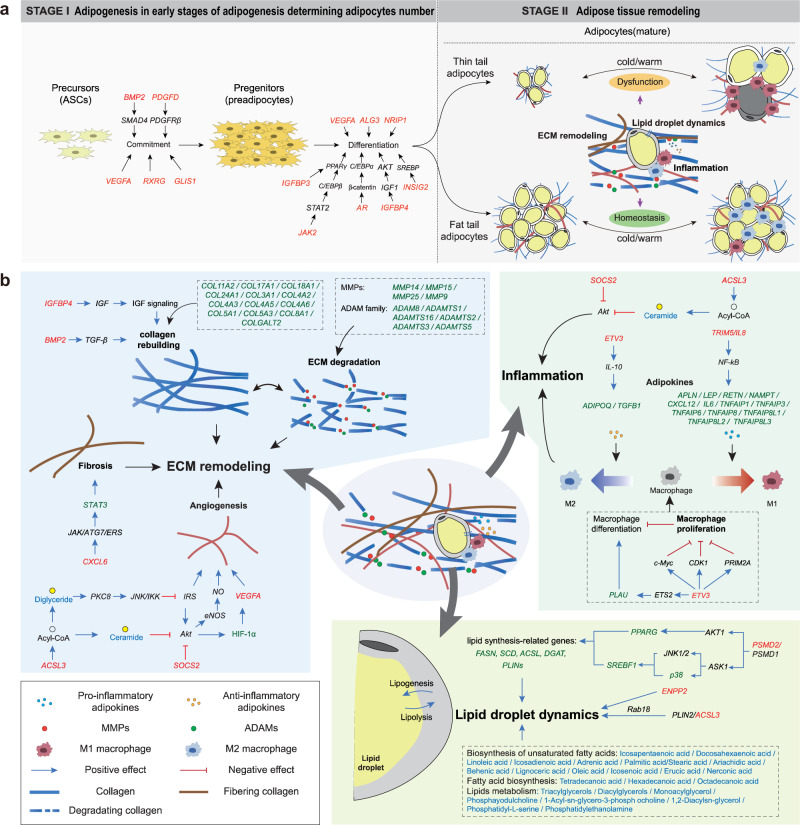
Schematic of the regulatory mechanisms of adipocytes in sheep in response to environmental changes. **a** Proposed two-phase regulatory mechanisms in tail adipose tissues of sheep with different fat deposition patterns. Phase I: genetic mutations influence adipocyte number in the early stages of adipogenesis; Phase II: the mature tail adipose tissues in fat-tailed and thin-tailed sheep regulate homeostasis in response to environmental changes. **b** The detailed regulatory process of homeostasis in mature adipose tissues at the genomic, transcriptomic, and lipidomic levels by ECM remodeling, lipid droplet dynamics, and inflammation. The candidate genes identified by whole-genome selective sweep, transcriptomic and lipidomic analyses are shown in red, green, and blue, respectively. The blue arrow indicates a positive effect, and the red arrow indicates a negative effect.

The 98 sweep regions contained 43,584 SNPs, and 31,798 of the SNPs showed significantly different frequencies between fat-tailed and thin-tailed populations under the Z-test. These 31,798 SNPs were annotated to be in or near 244 genes, and 97, 7840, 346, 634, and 22416 SNPs were located in exonic, intronic, UTR, up/downstream and intergenic regions, respectively (Supplementary Table 2). Functional annotation of these genes revealed 12 candidate genes associated with adipogenesis (e.g., *BMP2*, *PDGFD*, *IGFBP3*, *IGFBP4*, *GLIS1*, *ALG3*, *NRIP1*, *RXRG*, *JAK2*, *AR*, *INSIG2*, and *VEGFA*), five genes associated with ECM remodeling (*IGFBP4*, *BMP2*, *ACSL3*, *SOCS2*, and *VEGFA*), five genes associated with inflammation (*SOCS2*, *ACSL3*, *ETV3*, *TRIM5*, and *IL8*) and three genes associated with lipid droplet dynamics (*PSMD1*, *ENPP2*, and *ACSL3*) (Fig. 6). For the *BMP2*, *PDGFD*, and *VEGFA*, haplotypes of the SNPs in the selective regions around the three genes showed significant differences between fat-tailed and thin-tailed populations, and the most significant SNPs identified by Z-tests in the three regions were chr13:51,250,257 bp(G/A), chr15:4,172,397 bp (A/G), and chr20:19,040,316 bp (C/T) (Fig. 5e). GO and KEGG enrichment analyses of the 244 genes revealed their essential roles in response to lipid (e.g., *NRIP1*, *CXCL8*, and *AR*), cellular response to lipid (e.g., *TNFAIP3*, *PTK6*, and *ALDH1A2*), adipokine (e.g., *JAK2*, *ACSL3*, and *CAMKK2*) and transport-related pathways (e.g., *ABCC10*, *ABCD1*, and *NDC1*) (Fig. 5f and Supplementary Tables 3, 4).

## Discussion

Adipose tissue serves as an organ for energy storage, heat production, water supply, and thermal insulation in animals such as camel, African fat-tailed gecko, fat-tailed gerbil, fat-tailed dwarf lemur, fat-tailed dunnart, and fat-tailed sheep^39^, and plays a critical role in their ability to survive and thrive in extreme environments^40,41^. Histological examinations showed that the adipocyte size of tail fat increased in summer but decreased in winter, reflecting the energy storage and mobilization processes. Sheep largely rely on the energy stored in fat to survive cold and food shortages. From the growing season to the dormant season, fat-tailed sheep lose 18.85–26.05% and thin-tailed sheep lose 30.27–48.15% of their body weight^42–44^, suggesting that fat-tailed sheep have a larger energy reserve. Considering that the seasonal change in adipocyte size in the tail fat of fat-tailed sheep was smaller than that of thin-tailed sheep, the enlarged energy storage capacity of fat-tailed sheep is largely due to an increase in adipocyte number. With a large number of adipocytes, the adipocyte volume is less affected by nutritional and environmental challenges.

We observed tissue-specific DEGs for the five fat depots, which were significantly (*p*_adj._ < 0.05) enriched in different functional pathways. Intermuscular fat was more engaged in pathways related to mitochondria, ATP, metabolic processes, and biosynthetic processes than the other adipose tissues, which might contribute to the energy supply for muscle contraction. However, the DEGs in the tail fat showed their main functions in inflammatory reactions, cytokines, ECM regulation, transportation, and hemopoiesis processes in summer, which could be associated with their ability to rapidly expand tail fat in summer. These findings implied that tail fat and intermuscular fat have different biological functions in sheep. Intriguingly, the size of tail adipocytes decreases, whereas the size of intermuscular adipocytes increases in response to food shortages in winter. Intermuscular fat is a reservoir of heterotopic fat located in the subfascial and between muscle bundles, and it has been reported as an important predictor of muscle function and mobile function in human medical studies^38^.

As the main lipid storage depot, adipocytes play a crucial role in buffering the daily influx of dietary fat into circulation. In humans, body fat distribution is of clinical interest because of its links to multiple metabolic diseases^45^. Previous studies reported that fat stored in the trunk was more pathogenic than that stored in other compartments, and the accumulation of adipose tissue in the upper body (abdominal region) was more pathogenic than that in the lower body (gluteofemoral region)^46^. Lower body fat has a more beneficial inflammatory phenotype. In addition, body fat is typically classified into subcutaneous and visceral fat, and the latter is often associated with metabolic disease^47^. Thus, the increased adipocyte size in the intermuscular adipose tissues in winter might be linked to the inflammatory response caused by the cold resistance of muscle tissues in winter^48^.

Adipose tissue expansion occurs via hyperplasia and hypertrophy^49^. In fat-tailed sheep, the large tail fat mass is gained mainly through hyperplasia but not hypertrophy because the seasonal variation in adipocyte size was significantly smaller than that in thin-tailed sheep (Fig. 4a). Selective sweeps tests detected a set of genes involved in adipocyte hyperplasia, which have been identified as candidate genes for the fat tail phenotype of involvement in the sheep in previous studies, such as *BMP2*^5,19,20^, *PDGFD*^18,23,50,51^, *GLIS1*^16^, and *VEGFA*^52^. In addition, selective sweep tests detected a few candidate genes involved in terminal adipogenic differentiation such as *IGFBP-3* (Interference with PPARγ)^53^, *INSIG2* (Involvement in human adipocyte metabolism and body weight regulation)^54^, *JAK2* (The *JAK2/STAT3* pathway regulates C/EBPβ transcription)^55^, *ALG3* (Being involved in the biosynthesis of the N glycan precursor)^56^ and *Nrip1* (Deletion of *Nrip1* could decrease cell proliferation, prevent cell apoptosis, and suppress adipogenesis)^57^. These genes are involved in adipocyte hyperplasia and terminal adipogenic differentiation in the early stages of adipogenesis, resulting in a large difference in the adipocyte numbers in tail fat between fat-tailed sheep and thin-tailed sheep (Fig. 6a).

The number of adipocytes is mostly fixed in early life, while the size of adipocytes varies in response to the dynamic nutritional status in adulthood. Typically, the adipocytes of sheep are enlarged in summer because of an excessive energy supply but shrink in winter due to food deprivation or increased thermogenic activities. This process is called adipose tissue remodeling. Here, we observed that the tail fat of fat-tailed sheep exhibited advantages in maintaining energy homeostasis during seasonal changes. Although the mass of tail fat in fat-tailed sheep was much larger than that in thin-tailed sheep, fat-tailed sheep exhibited lower levels of inflammation. Overexpansion of adipocytes is metabolically unhealthy but the enlargement of tail adipocytes in fat-tailed sheep in summer is moderate compared to their size in winter, which is likely due to the genetically enhanced tail adipocyte hyperplasia. In addition, selective tests identified inflammation-related genes such as *ETV3*, *TRIM5*, *IL8*, and *SOCS2*, which might have contributed to the inflammatory profile difference in tail adipose tissues between fat-tailed sheep and thin-tailed sheep during the seasonal changes (Figs. 4g–i and 6b).

In addition, the functions of adipocytes are greatly affected by the ECM, a network of proteins and proteoglycans that controls cell differentiation, migration, repair, adhesion, and development^58^. Changes in adipocyte size are accompanied by ECM remodeling, which involves multiple processes such as collagen rebuilding, ECM degradation, fibrosis, and angiogenesis;^59^ these processes could be regulated by the candidate genes under selection in fat-tailed sheep such as *IGF-1, IGFBP4, BMP2, CXCL6, ACSL3, SOCS2*, and *VEGFA* (Fig. 6b). Consistent with their greater extent of adipocyte enlargement, thin-tailed sheep had higher expression of genes involved in ECM remodeling because of the greater demand for reshaping adipocytes and their extracellular environment to deal with the excess energy supply in summer.

Adipose tissue is an important regulator of energy balance and nutritional homeostasis. Here, we detected genes and lipids that were involved in lipid droplet dynamics at the genomic, transcriptomic, and lipidomic levels. The selected genes included *PSMD1*, *ENPP2*, and *ACSL3*, which were previously reported to be related to lipid synthesis-related genes and lipid droplet dynamics^60,61^. Correspondingly, the downstream genes regulated by these genes showed different expression patterns between fat-tailed and thin-tailed sheep. Many lipids were involved in biological processes, such as biosynthesis of unsaturated fatty acids, fatty acid biosynthesis, and lipid metabolism, which together regulated the lipid droplet dynamics (Fig. 6b).

By integrating the genomic, transcriptomic, and lipidomic data, we observed homeostasis in the tail fat of fat-tailed sheep. Adipose tissues, the most dynamic organ in adults^62^, play a crucial role in energy and metabolic homeostasis, and they expand or shrink according to nutrient status. Healthy adipose tissue expansion is associated with enhanced adipogenesis, decreased inflammation, and minimized fibrotic damage, which is called homeostasis. However, dysfunctions in these aspects typically result in an unhealthy pattern of adipose tissue expansion^63^. In this study, the adipocyte size of adipose tissue in thin-tailed sheep decreased sharply from summer to winter, while the adipose tissue of fat-tailed sheep maintained stability (Fig. 4a). Therefore, the adipose tissue of fat-tailed sheep was morphologically more stable than the adipose tissue of thin-tailed sheep. We concluded that the dramatic increase in adipocyte number in the tail fat depot of fat-tailed sheep is coupled with an ability to regulate ECM remodeling, inflammation, and lipid dynamics in adipose tissues (Fig. 6b). Furthermore, the expression of genes in these pathways was more stable in the tail adipose tissues of fat-tailed sheep during the season change than in those of thin-tailed sheep (Fig. 4d–g). Therefore, we hypothesized that in addition to genes regulating adipocyte/precursor numbers in the early stages of adipogenesis, genes that regulate ECM remodeling, lipid synthesis and degradation, and immune reactions are also important in regulating the ability of the tail fat of sheep to adapt to seasonal food instability.

In conclusion, we established transcriptome and lipid profiles of adipose tissues from different fat depots of fat- and thin-tailed sheep populations in summer and winter. We found that the evolutionary mechanisms of fat-tailed sheep involved not only in the early stages of adipogenesis, but also later ECM remodeling, inflammation, and lipid droplet dynamics in mature adipose tissue in response to seasonal climate changes. Our findings provide insights into the stress resistance mechanism of fat-tailed sheep. In addition, the candidate genes related to the tail phenotype provide a genomic foundation for future genetic improvement of fat-tailed sheep.

## Methods

### Adipose tissue collection

We selected 50 ewes (2–3 years old) of Tibetan sheep, Chinese Merino, Small-tailed Han, Wadi, and Altay sheep, representing the typical short-thin-tailed, long-thin-tailed, short-fat-tailed, long-fat-tailed, and fat-rumped sheep populations, respectively (Fig. 1a). In the five populations, we collected adipose tissues from five different body fat depots (subcutaneous, tail, perirenal, intermuscular, and caul fat) for RNA-Seq and lipid profiling, consisting of 25 animals in summer (June–August) and 25 animals in winter (November–January). For each tissue, we included five independent samples from five different individuals in each population, totaling 250 samples in the five populations (Fig. 1b, c and Supplementary Data 1). Caul fat (greater omentum) was stripped from the preperitoneal adipose tissue of the abdominal wall. Perirenal fat was obtained from the fat layer surrounding the kidneys. Intermuscular fat was stripped from fat deposits between the hind leg muscles. Subcutaneous fat was obtained from the subcutaneous fat layer on the shoulder of the sheep. Tail fat was sampled from the subcutaneous fat layer of sheep tails. The tissue samples were immediately stored in liquid nitrogen after dissection from the animal’s body within half an hour after death, and kept in liquid nitrogen for more than 30 minutes. The samples were then transferred to a −80 °C freezer until RNA extraction. All animal operations were conducted according to the guidelines and regulations approved by the Institutional Animal Care and Use Committee of China Agricultural University (CAU20160628-2) and the local animal research ethics committee.

### RNA-Seq data

#### RNA extraction, library preparation, and sequencing

Total RNA was extracted from the adipose tissues with the RNA TRIzol (TaKaRa, USA) according to the manufacturer’s protocol. The quality and concentration of RNA were evaluated with the Agilent 2100 RNA 6000 Nano Kit (Agilent Technologies, Waldbronn, Germany). RNA-Seq libraries were constructed with the NEB Next Ultra^TM^ RNA Library Prep Kit (New England Biolabs, Ipswich, MA, USA) and sequenced using the Illumina Novaseq 6000 (Illumina, San Diego, USA) with a 150 bp paired-end protocol. Each library was sequenced with a minimum data output of 6 Gb (Supplementary Data 2).

#### RNA-Seq data quality control

Raw RNA-sequencing (RNA-seq) reads were first trimmed using fastp v0.20.1^64^ with the following setting parameters: (1) over 50% of bases with low base quality scores (≤ 20) or (2) a missing rate of bases more than 10%. The trimmed RNA-Seq reads were then mapped against the sheep reference genome *Oar_Rambouillet v1.0* (https://www.ncbi.nlm.nih.gov/assembly/GCF_002742125.1) using Hisat2 v2.1.0^65^ with the default setting. Afterwards, the counts of known protein-coding genes were quantified with the function featureCounts and normalized using the transcripts per million (TPM) method in the Subread package v2.0.3^66^.

Furthermore, the quality and comparability of the sequencing libraries were examined using unsupervised hierarchical clustering and principal component analysis (PCA) approaches with the hclust function in the R packages stats v 4.1.0^67^ and FactoMineR v 4.1.2^68^. Outliers or potentially contaminated samples were excluded in the following analyses.

### Lipidomic data

#### Lipid extraction

For all 250 adipose tissues, lipids were extracted using the methyl-tert-butyl ether (MTBE) method^69^. In brief, 20 mg of adipose tissues were thawed on ice and homogenized in 1 ml of methanol/MTBE (1:3, v/v, with internal standard added) for 15 min on a roller mixer (210 rpm). Afterward, 200 μl of ddH_2_O was added to the mixture, incubated for 1 min on a roller mixer (210 rpm), and then centrifuged at 12,000 rpm at 4 °C for 10 min to achieve phase separation. Subsequently, 300 μl of the supernatant was extracted and dried in an oven, dissolved in 200 μl of mobile phase B and stored at −80 °C until later analyses. The dissolved solution was put into the sample bottles for the subsequent LC-MS/MS analysis.

#### Lipid profiling, identification, and quantification

Lipid profiling, identification, and quantification were implemented as described below, following the protocol from a previous study^70^. In more detail, the lipid profiling was first conducted with a LC-ESI-MS/MS system, which combines the ultra performance liquid chromatography (UPLC) using the SCIEX ExionLC AD system (Danaher Corporation, America) and the tandem mass spectrometry (MS/MS) using the SCIEX QTRAP ® LC-MS/MS system (Danaher Corporation, America).

The UPLC analytical conditions were as follows: UPLC: column, Thermo Accucore™ C30 (2.6 μm of length, 100 bores 2.1 mm in diameter); solvent system, A: acetonitrile/water (60/40, V/V, 0.1% formic acid, 10 mmol/L ammonium formate), B: acetonitrile/isopropanol (10/90, V/V, 0.1% formic acid, 10 mmol/L ammonium formate). The stepwise gradient elution was set as follows: A/B (80:20, V/V) at 0 min, 70:30 V/V at 2.0 min, 40:60 V/V at 4 min, 15:85 V/V at 9 min, 10:90 V/V at 14 min, 5:95 V/V at 15.5 min, 5:95 V/V at 17.3 min, 80:20 V/V at 17.3 min, 80:20 V/V at 20 min; flow rate, 0.35 ml/min; temperature, 45 °C; and injection volume, 2 μl. The effluent was alternatively connected to an ESI-triple quadrupole-linear ion trap (QTRAP)-MS.

The combined linear ion trap (LIT) and triple-quadrupole (QqQ) scans were acquired on a QTRAP® LC-MS/MS System, which was equipped with an ESI Turbo Ion-Spray interface, operated in positive and negative ion mode and controlled by Analyst 1.6.3 software (Sciex, Framingham, MA). The ESI source operation parameters were as follows: ion source, turbo spray; source temperature, 500 °C; ion spray voltage (IS), 5500 V (positive), −4500 V (negative); ion source gas 1 (GS1): 45 psi, gas 2 (GS2): 55 psi, curtain gas (CUR): 35 pis; collision gas (CAD), medium. Instrument tuning and mass calibration were performed with 10 and 100 μmol/L polypropylene glycol solutions in QqQ and LIT modes, respectively. QqQ scans were acquired as multiple reaction monitoring (MRM) experiments with the collision gas (nitrogen) set to 5 psi. Declustering potential (DP) and the collision energy (CE) for individual MRM transitions were determined with further DP and CE optimization. A specific set of MRM transitions was monitored for each period according to the metabolites eluted within this period.

### Whole-genome sequence data

#### Data collection

Whole-genome sequences of 525 domestic sheep (average depth = ~16×) were retrieved from six sources: the NextGen Consortium and five previous studies^15,71–74^, including 221 fat-tailed sheep from 46 populations (9 fat-rumped, 31 fat-tailed, 3 long fat-tailed, and 3 short fat-tailed populations), and 304 thin-tailed sheep from 54 populations (1 long thin-tailed, 23 short thin-tailed, and 30 thin-tailed populations) (Fig. 5a and Supplementary Data 35). Detailed information on the populations, including the names, sampling locations, number of samples, and tail morphology, are provided in Supplementary Data 35.

#### Variant calling

SNP calling was implemented following the sequential protocols of a previous study^73^. In summary, six procedures were implemented as follows: (1) low-quality bases and artifact sequences were processed and removed using Trimmomatic v 0.36^75^; (2) the high-quality 150-bp/100-bp paired-end reads were aligned to the sheep reference genome *Oar_rambouillet_v1.0*. (https://www.ncbi.nlm.nih.gov/assembly/GCF_002742125.1/) using the Burrows‒Wheeler aligner (BWA mem) v0.7.8^76^ with default parameters; (3) the bam files obtained were processed by the MarkDuplicates module in GATK v4.1.2.0^77^ to remove duplicates; (4) short variations (SNPs and indels) were detected by the GATK *HaplotypeCaller* module under the GATK best-practice recommendations^77^; (5) the merging of GVCFs files called individually and SNP calling were implemented by the *CombineGVCFs* module and the *GenotypeGVCFs* module, respectively; and (6) the raw SNPs were selected by the *SelectVariants* module in GATK and filtered using “VariantFiltering” of the GATK under the parameters “QUAL < 30.0 || QD < 2.0 || MQ < 40.0 || FS > 60.0 || SOR > 3.0 || MQRankSum < −12.5 || ReadPosRankSum < −8.0”.

#### SNP quality control

We filtered the SNP dataset using VCFtools v0.1.17^78^. SNPs that met any of the following conditions were removed: (1) call rate ≤ 90%; (2) minor allele frequency (MAF) ≤ 0.05; or (3) mean max depth <3 or >30. After quality control, 27,412,352 SNPs were retained for the selective sweep tests.

#### Population genetics analysis

To examine the genetic differentiation of 525 domestic sheep, we performed PCA, phylogenetic tree, and structure analyses. We extracted independent SNPs using LD pruning with the PLINK option “–indep-pairwise 50 5 0.2”. Finally, a total of 1,179,613 SNPs were retained for the following analyses. We performed PCA analysis using the smartpca model in the EIGENSOFT v.6.0.1^79^ under the default settings. To build a phylogenetic tree, the genetic distance matrix between the individuals was calculated using the PLINK v1.90 (–distance 1-ibs), and an unrooted neighbor-joining (NJ) tree was constructed using the SplitsTree v4.17.1^80^ and visualized by Figtree v1.4.4 (http://tree.bio.ed.ac.uk/). Furthermore, model-based clustering was carried out using the sNMF v1.2^81^ with the number of cluster *K* from 2 to 9.

### Transcriptome analysis

#### Identification of differentially expressed genes (DEGs)

For the tissues from the same fat depot, we performed comparisons to identify genes that were differentially expressed in each population between winter and summer (Supplementary Data 3). For each population in the same season, differential expression analyses were conducted between adipose tissues from different fat depots (Supplementary Data 10). All differential expression analyses were implemented using DESeq2 v3.12 (Love et al., 2014) with default parameters. The fold change values were estimated based on the normalized gene expression level in each sample. A false discovery rate (*p*_adj_) < 0.05 and an absolute value of log_2_(fold change value) ≥ 1 were used as the significance thresholds.

#### Weighted gene correlation network analysis (WGCNA)

To characterize the global transcriptional responses of the tail adipose tissues to seasonal change, weighted gene coexpression analysis (WGCNA) was performed between fat-tailed and thin-tailed sheep populations using the WGCNA package in R (Langfelder and Horvath, 2008). Briefly, we first normalized the matrix of read counts with the variance stabilizing transformation (VST) procedure in DESeq2 v.3.12. The normalized matrix was then transformed into an adjacency matrix step by step. Genes with similar expression patterns were clustered into 15 distinct modules. We calculated the Pearson’s correlation of the 15 modules with four groups of tail-season combinations (summer fat-tail, summer thin-tail, winter fat-tail, and winter thin-tail).

#### Gene ontology (GO) and pathway enrichment analyses

Genes from correlated WGCNA modules were used to generate and visualize networks based on GO-enrichment analysis (GOEA) using the ClueGo and Word Clouding plugins in Cytoscape software^82,83^. GO term and KEGG pathway enrichment analyses of the DEGs were conducted with the ClusterProfiler package in the R program^84^. GO terms and KEGG pathways were defined to be significantly enriched under the adjusted threshold of *p* value < 0.05.

### Lipidomic analysis

#### PCA of lipid composition

The data were scaled with unit variance, and then unsupervised PCA was performed using the function prcomp in R v4.1.0. Hierarchical cluster analysis (HCA) was implemented using the function hclust in R v4.1.0. The HCA results of samples and metabolites were depicted in heatmaps and dendrograms, while the Pearson correlation coefficients (PCCs) among samples were calculated using the cor function in R v4.1.0 and visualized in heatmaps. The results of HCA and PCC were visualized by the R package ComplexHeatmap v1.20.2^85^. For HCA, the normalized signal intensities of metabolites using the unit variance scaling were visualized as a color spectrum.

#### Differential metabolite selection

We identified significantly regulated metabolites between groups with the criteria of variable importance in a project (VIP) ≥ 1 and absolute Log_2_FC (fold change) ≥ 1. We extracted the VIP values from the orthogonal projections to latent structures discriminant analysis (OPLS-DA) results, which contained score plots and permutation plots and were generated using the R package MetaboAnalystR 3.0^86^. Before OPLS-DA, the data were log-transformed (log_2_) and mean-centered. Then, a permutation test with 200 permutations was performed to avoid overfitting.

#### KEGG annotation and enrichment analysis

The metabolites identified above were first annotated using the Kyoto Encyclopedia of Genes and Genomes (KEGG) Compound database (http://www.kegg.jp/kegg/compound/). Annotated metabolites were then mapped to the KEGG pathway database (http://www.kegg.jp/kegg/pathway.html). For a given list of metabolites, enriched pathways were identified to be as significant with an adjusted *p*-value < 0.05 by a minimum hypergeometric test.

### Whole-genome selective sweep tests

To identify potential selective signatures between fat-tailed and thin-tailed populations, three methods were implemented to scan the genomes for selective sweeps. We calculated the genome-wide pairwise *F*_ST_ values^87^ and *−*log_2_(π_fat-tailed_/π_thin-tailed_) values with a slide-window approach (50 kb sliding windows with 25 kb steps) with the program VCFtools v0.1.17^78^, and conducted the cross-population composite likelihood ratio test (XP-CLR) in 50-kb sliding windows with a 25-kb step size using the program XP-CLR v1.0^88^. We compared the profiles of EHH between the fat-tailed population and thin-tailed population by calculating XP-EHH statistics using Selscan 2.0.0 software^89^ in 50-kb sliding windows with a 25-kb step size.

### Histological examination and validation of macrophage markers

#### Gene expression validation of macrophage markers

Reverse transcription quantitative real-time PCR (RT‒qPCR) was used to validate the gene expression levels of M1 macrophage markers (*TNF* and *NOS2*) and M2 macrophage markers (*MRC1* and *CD40*) in tail adipose samples including 15 summer fat-tail adipose tissues, 10 summer fat-tail adipose tissues, 15 winter fat-tail adipose tissues, and 10 winter fat-tail adipose tissues. Total RNA was isolated from adipose tissues with TRIzol (TaKaRa, USA) according to the manufacturer’s protocol. cDNA was generated from total RNA using the RevertAid First Strand cDNA Synthesis Kit (Thermo Fisher Scientific, Waltham, MA, USA) according to the manufacturer’s instructions. Subsequently, qPCR (Quantitative PCR) was performed on the QuantStudio 1 Real-Time PCR System (Life Technologies, Carlsbad, CA, USA) for 4 macrophage makers genes and 11 genes related to ECM remodeling, lipid droplet dynamic and inflammation with the designed primers (Supplementary Table 1). Relative expression levels of the markers were measured using the 2^−ΔΔCt^ method with 18S rRNA (forward: 5′-CCTGCGGCTTAATTTGACTC; reverse: AACTAAGAACGGCCATGCAC-3′) as the endogenous reference. qPCR was performed in 20 μl volume consisting of 10 μl 2× SYBR Green mix (Applied Biosystems, Foster City, CA, USA), 2 μl cDNA, 2 μl (10 pmol/μL) reverse and forward primers, and 4 μl ddH_2_O using the following cycling parameters: 2 min at 50 °C, 2 min at 95 °C (heat activation step) and 40 cycles of 15 s at 95 °C and 1 min at 60 °C. Dissociation curve analyses were performed using the instrument’s default setting immediately after each PCR run. Each qPCR was run 3 times for one sample as technical replicates. Statistical analysis was performed using ANOVA with Duncan’s test in SPSS software (version 10.0; SPSS, Chicago, IL).

#### Tissue processing and histological examination

Adipose tissues for histological analysis were fixed in 4% Paraformaldehyde (PFA) Solution for 24 hours, embedded in paraffin, then sectioned into 5 μm thick sections. For adipocyte size, tissue sections were stained with hematoxylin and eosin^90^ and imaged under an EVOS XL Core microscope. The size of adipocytes was measured (3 images per tissue) using ImageJ (NIH). For immunofluorescence (IF) staining, representative slides were selected according to the average adipocyte size. Tissue sections were heated in citrate buffer (10 mM citric acid, 0.05% Tween 20, pH 6.0) at 95~100 °C for 20 minutes for antigen retrieval. Sections were then blocked with 5% goat serum in TBS containing 0.3% Triton X-100 for 1 h, incubated with mouse F4/80 antibody (1:500 dilution) (ABclonal, Cat. No. A18637) at 4 °C for 12 h, washed in PBS 5 times and then incubated with secondary antibody at room temperature for 1 h. Sections were mounted in Fluoroshield mounting medium with DAPI (ab104139, Cambridge, MA). Immunofluorescence images were captured under an EVOS fluorescence microscope (ThermoFisher Scientific, MA, USA).

### Statistics and reproducibility

The statistical tests used in this study were performed using R v4.1.0 or SPSS v10.0, and details statistical analyses were described within the methods section. Differentially expressed genes were identified using log_2_(fold change value) ≥ 1 and FDR-adjusted *P*-values(*P*_adj_ < 0.05). Significantly GO terms and KEGG pathways were defined under the adjusted threshold of *P*-value < 0.05. For qPCR experiments, at least three biologically independent replicates were adopted and three technical replicates for each sample were run, statistical analysis was performed using ANOVA with Duncan’s test in SPSS software. Adipocyte diameter between summer and winter was measured using a two-tailed Student’s *t* test. The significant *P*-values were marked with *P < 0.05, **P < 0.01, and ***P < 0.001.

### Reporting summary

Further information on research design is available in the Nature Portfolio Reporting Summary linked to this article.

## Supplementary information

Supplementary Information

Description of Additional Supplementary Files

Supplementary Data 1-45

Reporting Summary

## Data Availability

All raw RNA-seq data generated in this study were deposited in NCBI’s SRA under the accession number PRJNA909968. Additional source data for Figs. 2, 3, 4a–c, h are provided as Supplementary Data 3–5, 10–14, and 19–33.

## Code availability

All scripts used for this work were performed using open source software tools, and are available from the corresponding author upon request.
