# Supplementary material for: Whole-body adipose tissue multi-omic analyses in sheep reveal molecular mechanisms underlying local adaptation to extreme environments
Source: Commun Biol. 2023 Feb 8;6:159. doi: 10.1038/s42003-023-04523-9 (PMC9908986; doi:10.1038/s42003-023-04523-9)
Supplement: Supplementary file 1 — Supplementary Information [file 42003_2023_4523_MOESM1_ESM.pdf]

Supplementary Information for

Whole-body adipose tissue multi-omic analyses in sheep reveal molecular mechanisms underlying local adaptation to extreme environments

Ya-Xi Xu, Bo Wang, Jia-Nan Jing, Rui Ma, Yan-Hui Luo, Xin Li, Ze Yan, Ya-Jing Liu, Lei Gao, Yan-Ling Ren, Meng-Hua Li*, Feng-Hua Lv*

*To whom correspondence may be addressed. E-mail:

lvfenghua@cau.edu.cn (Feng-Hua Lv)
menghua.li@cau.edu.cn (Meng-Hua Li)

This PDF file includes:

Supplementary Tables 1-4
Supplementary Figures 1-21

Supplementary Table 1. Primers used for RT-qPCR.

Functions	Genes	Accession no.	Forward primers	Reverse primers	Product length
M1/M2 macrophage makers	*TNF*	NM_001024860.1	GGCCTTTGTGCCTCCTTTTG	GGTTACAGGCATGACTCCCC	137 bp
	NOS2	XM_042255454.1	AGACTGAGCCTCTCTAGCCC	GGAACCGTCTATAGCTGCCC	96 bp
	MRC1	NM_001197180.1	CGGATATGCCAGGACGGAAA	CTTCTGCCCAGTGTTTGCAC	120 bp
	CD40	XM_012189293.4	AATGCCAGTCCTGCGGTAAA	CCAAAACCCCTGTAGCGATCT	246 bp
ECM remodeling	*COL18A1*	XM_042237849.1	GTCCCGGGATGAATGGACTG	GTCCACAAATGCGTTGCTGT	152 bp
	MMP14	NM_001166181.1	AGGCCTTCCATCCCCGATAA	CAGAACCAACGCTCCTTGAA	122 bp
	MMP15	XM_027977892.2	TGGCTCTTCCGAGAAGCAAA	GAAGCGCCAATACCTGTCCT	153 bp
	ADAMTS1	XM_004002797.5	AGTTACCAGTGCAAGACCCG	GCCAGGAAGCTCCCGTTATT	117 bp
	STAT3	XM_015098790.3	AAAGACTCCGGGGATGTTGC	CAATCAGGGAGGCGTCACAA	187 bp
lipid droplet dynamics	*FASN*	XM_027974304.2	AACAAGACAAGCCCGAGGAG	AGGCCCAATTTCCTGGGATG	222 bp
	DGAT1	NM_001110164.1	GTGAGCTACCCCGACAACC	CGGGAGTAGTCCATGTCCTTG	233 bp
	PPARG	XM_027957558.2	TATTCTCAGTGGAGACCGCC	CTGCCTGAGGTCCGTCATTT	160 bp
	SREBF1	XM_027974786.2	GCACCGAGGCCAAGTTGAAT	ATGGGCACATCTGTGCTACC	187 bp
Inflammation	*ADIPOQ*	NM_001308565.1	TTCCACACCTGAGGGACTC	AGGACCAACGAGACCTGGAT	235 bp
	LEP	XM_027968780.2	ATCTCACACACGCAGTCCG	CTGGCGAGGATCTGTTGGTA	131 bp
Reference	*18S*	XM_047442803.1	CCTGCGGCTTAATTTGACTC	AACTAAGAACGGCCATGCAC	118 bp

Supplementary Table 2. Summary information of significant SNP variants (Z-test) under selective regions detected by at least two of the three methods based on the F_{ST}, π ratio, and XP-CLR estimates.

SNP variation region	SNP variants	No. of SNPs
Exonic region	Exonic (nonsynonymous SNV)	192
	Exonic (synonymous SNV)	180
	Exonic (unknown)	18
	Exonic (splicing)	1
	ncRNA_exonic	94
Intronic region	ncRNA_intronic	1,368
	intronic	6,472
UTR region	3' UTR	238
	5' UTR	108
Upstream/downstream	Upstream/downstream	634
Intergenic	Intergenic	22,416
Unkown	Unknown	77
In total		31,798

Supplementary Table 3. GO terms enriched with genes around SNPs significantly associated with the tail types.

Ontology	GO IDs	Description	GeneRatio	BgRatio	*P*-value	Count	Genes
BP	GO:0048565	digestive tract development	4/69	21/4727	2.06E-04	4	*CXCL8/ALDH1A2/PTK6/YIPF6*
BP	GO:0055123	digestive system development	4/69	22/4727	2.50E-04	4	*CXCL8/ALDH1A2/PTK6/YIPF6*
BP	GO:0048384	retinoic acid receptor signaling pathway	3/69	10/4727	3.32E-04	3	*RXRG/LOC101110922/ALDH1A2*
BP	GO:0033993	response to lipid	8/69	126/4727	4.36E-04	8	*NRIP1/LOC101110922/CXCL8/ALDH1A2/TNFAIP3/CCR7/PTK6/AR*
BP	GO:0071396	cellular response to lipid	7/69	96/4727	4.41E-04	7	*NRIP1/LOC101110922/CXCL8/ALDH1A2/TNFAIP3/PTK6/AR*
BP	GO:0009954	proximal/distal pattern formation	3/69	11/4727	4.52E-04	3	*LOC101110922/HOXC10/ALDH1A2*
BP	GO:0071300	cellular response to retinoic acid	3/69	11/4727	4.52E-04	3	*LOC101110922/ALDH1A2/PTK6*
CC	GO:0016591	RNA polymerase II, holoenzyme	5/76	30/5823	3.66E-05	5	*POLR2H/POLR2M/GTF2A1/PEX2/TAF1*
CC	GO:0000428	DNA-directed RNA polymerase complex	5/76	38/5823	1.19E-04	5	*POLR2H/POLR2M/GTF2A1/PEX2/TAF1*
CC	GO:0030880	RNA polymerase complex	5/76	38/5823	1.19E-04	5	*POLR2H/POLR2M/GTF2A1/PEX2/TAF1*
CC	GO:0055029	nuclear DNA-directed RNA polymerase complex	5/76	38/5823	1.19E-04	5	*POLR2H/POLR2M/GTF2A1/PEX2/TAF1*
CC	GO:0061695	transferase complex, transferring phosphorus-containing groups	5/76	61/5823	1.12E-03	5	*POLR2H/POLR2M/GTF2A1/PEX2/TAF1*
MF	GO:0003700	DNA-binding transcription factor activity	11/77	219/5491	2.07E-04	11	*ETV3/RXRG/HOXC6/HOXC9/HOXC10/RFX4/NR3C1/TBXT/ZGPAT/BHLHE40/AR*
MF	GO:0140110	transcription regulator activity	12/77	298/5491	7.98E-04	12	*ETV3/RXRG/NRIP1/HOXC6/HOXC9/HOXC10/RFX4/NR3C1/TBXT/ZGPAT/BHLHE40/AR*

Supplementary Table 4. KEGG pathways enriched with genes around SNPs significantly associated with tail types.

Pathway IDs	Description	GeneRatio	BgRatio	*P*-value	Count	Genes
oas03020	RNA polymerase	3/124	32/9099	9.19E-03	3	*POLR2H/POLR3B/POLR2M*
oas04061	Viral protein interaction with cytokine and cytokine receptor	5/124	97/9099	1.03E-02	5	*CXCL8/CXCL6/PPBP/CCR7/IL2RG*
oas02010	ABC transporters	4/124	63/9099	1.05E-02	4	*LOC101106534/LOC101106781/ABCC10/ABCD1*
oas04924	Renin secretion	4/124	71/9099	1.58E-02	4	*CLCA4/LOC101116002/LOC101116267/ITPR1*
oas04920	Adipocytokine signaling pathway	4/124	73/9099	1.74E-02	4	*RXRG/JAK2/ACSL3/CAMKK2*
oas05219	Bladder cancer	3/124	41/9099	1.81E-02	3	*CXCL8/CDH1/VEGFA*
oas04060	Cytokine-cytokine receptor interaction	10/124	356/9099	2.33E-02	10	*THPO/CXCL8/CXCL6/PPBP/CCR7/BMP2/TNFRSF6B/CXCL17/EDA2R/IL2RG*
oas01521	EGFR tyrosine kinase inhibitor resistance	4/124	83/9099	2.65E-02	4	*JAK2/NF1/PDGFD/VEGFA*
oas03013	Nucleocytoplasmic transport	5/124	124/9099	2.70E-02	5	*NDC1/SUMO2/NUP85/IPO9/EEF1A2*
oas03022	Basal transcription factors	3/124	48/9099	2.74E-02	3	*GTF2A1/TAF2/TAF1*
oas04657	IL-17 signaling pathway	4/124	92/9099	3.67E-02	4	*CXCL8/CXCL6/TNFAIP3/ANAPC5*

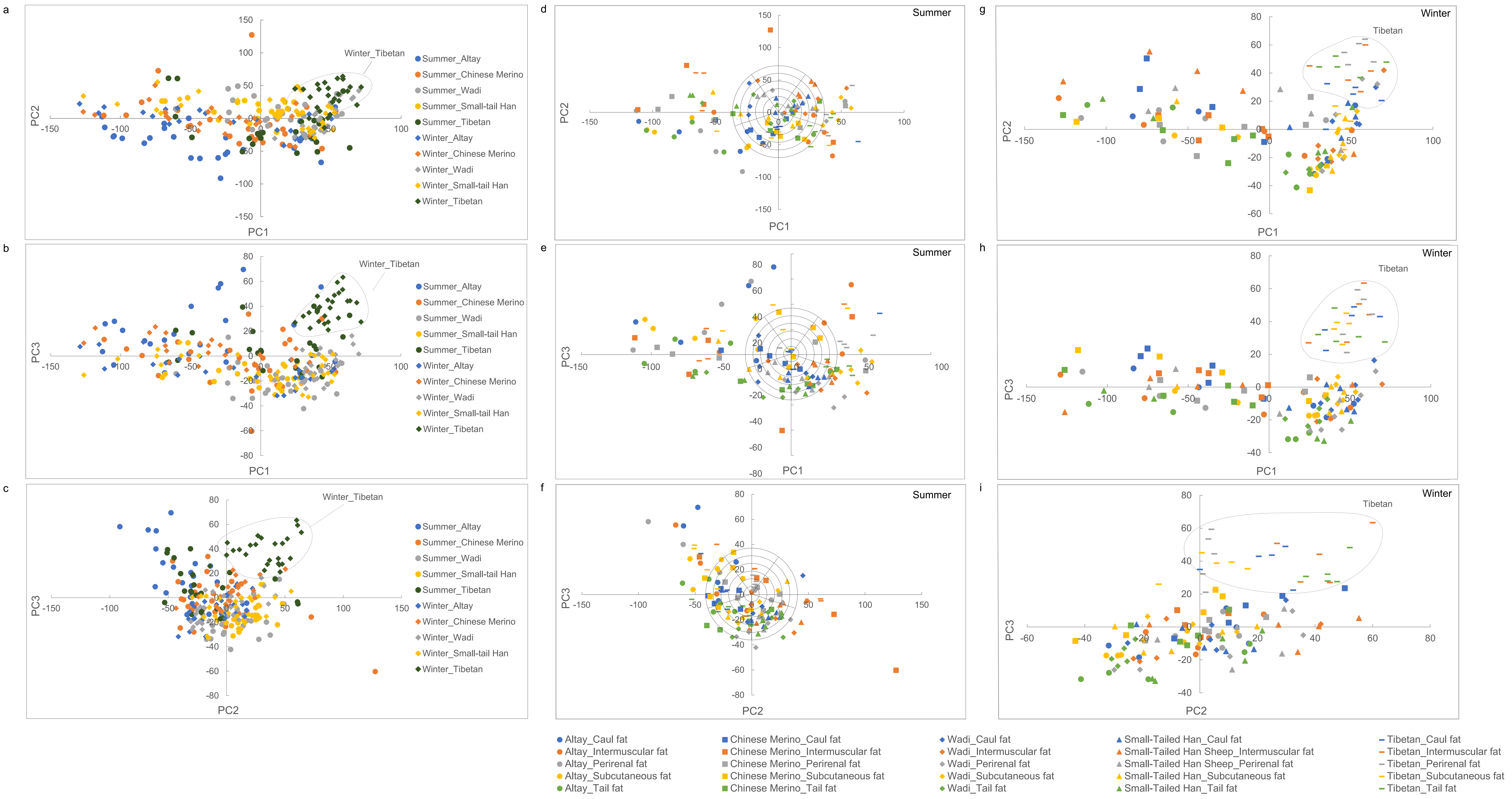

Supplementary Figure 1. Principal component analysis of gene expression. Scatterplots of PC1 versus PC2, PC1 versus PC3, and PC2 versus PC3 for 250 adipose tissues (a-c), 125 adipose tissues sampled in summer (d-f), and 125 adipose tissues sampled in winter (g-i). Points represent the RNA-Seq samples.

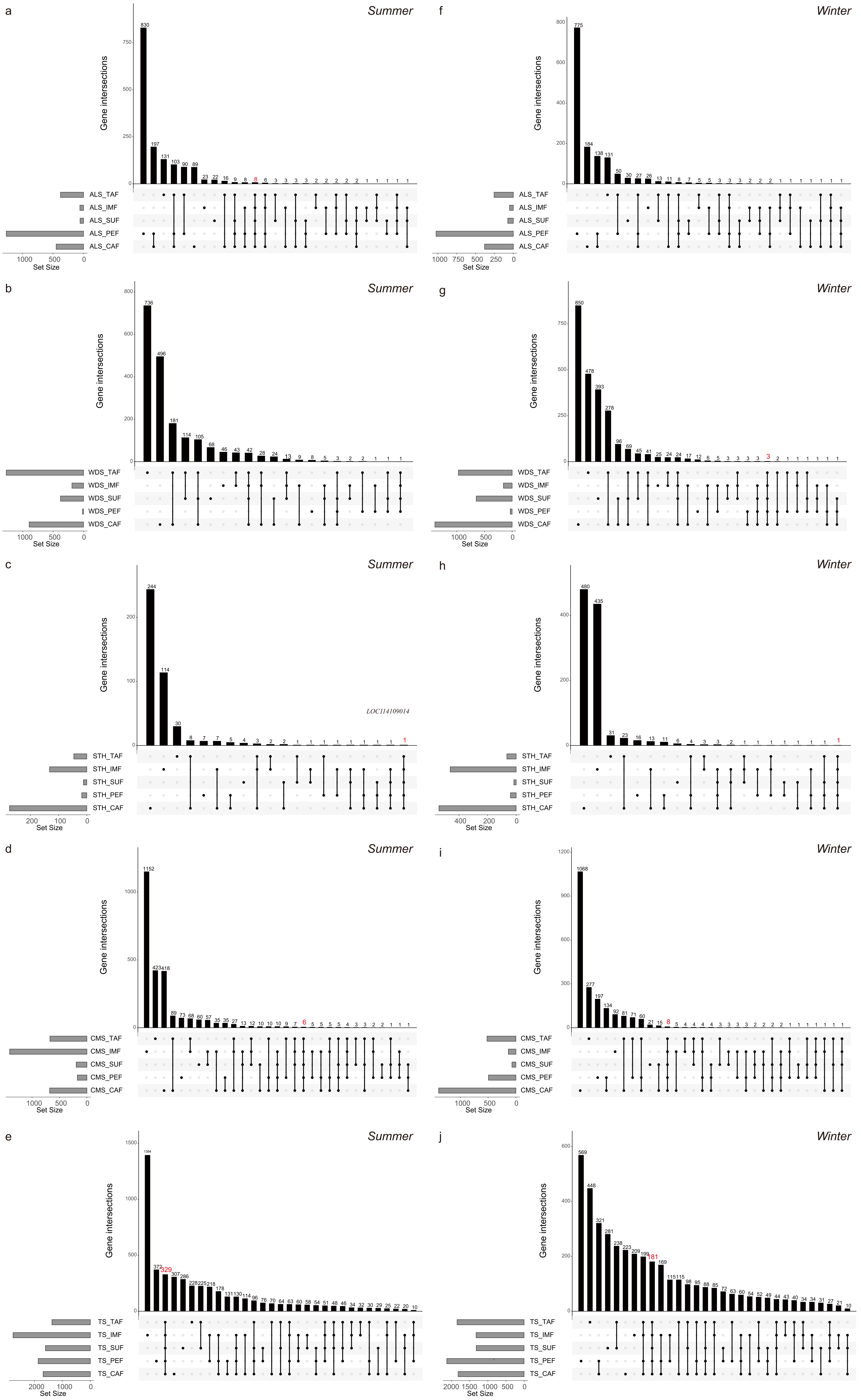

Supplementary Figure 2. UpSet plot of overlapping and depot-specific differentially expressed genes (DEGs) across the five adipose tissues of the five populations in summer and winter. a-e. The number of overlapping and depot-specific upregulated DEGs across the five adipose tissues of Altay sheep (a), Wadi sheep (b), Small-tailed Han sheep (c), Chinese Merino sheep (d), and Tibetan sheep (e) in summer. f-j. The number of overlapping and depot-specific upregulated DEGs across the five adipose tissues of Altay sheep (f), Wadi sheep (g), Small-tailed Han sheep (h), Chinese Merino sheep (i), and Tibetan sheep (j) in winter.

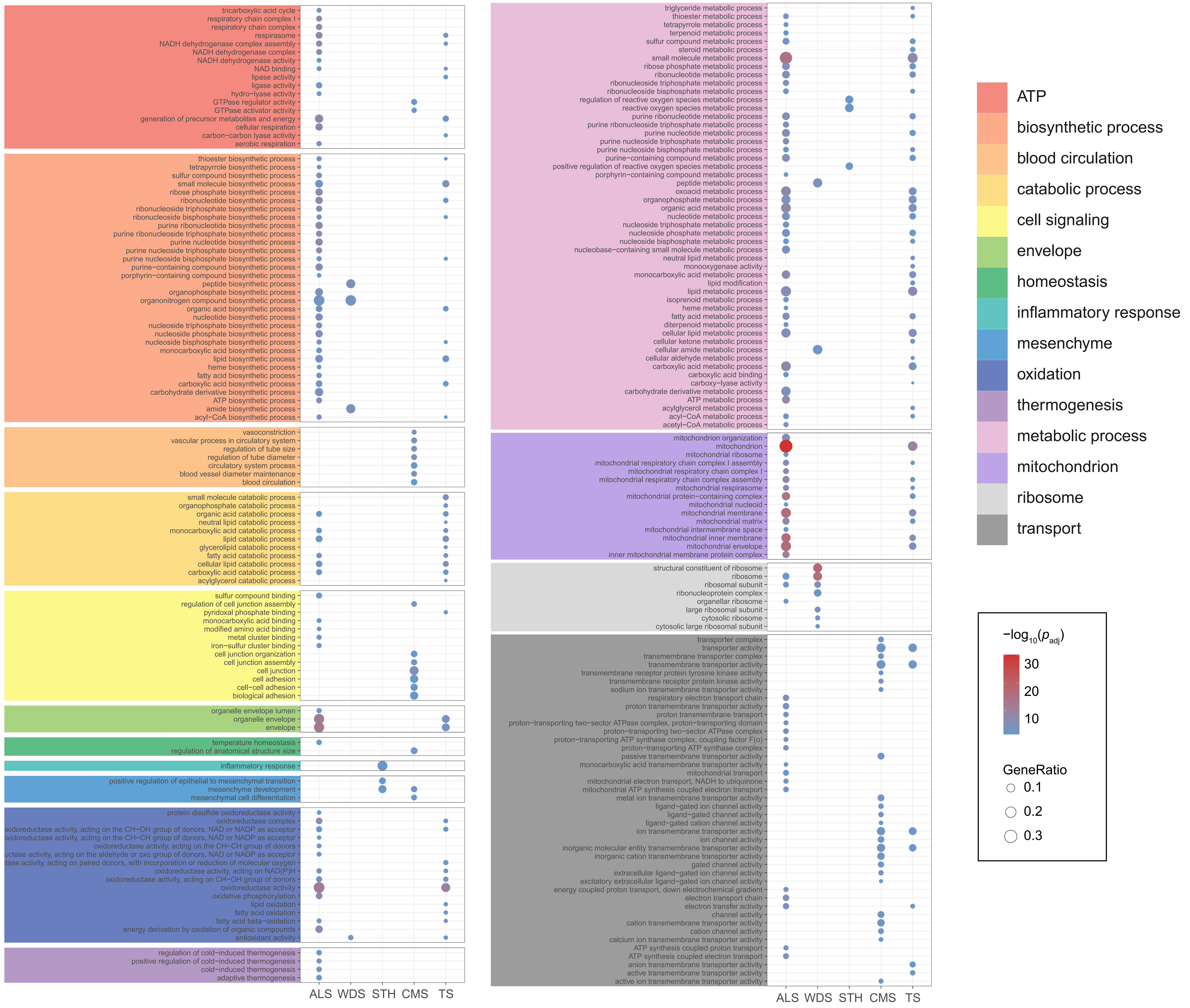

Supplementary Figure3. Significantly (*P*adj. < 0.05) enriched gene ontology (GO) terms for the differentially expressed genes (DEGs) upregulated in the adipose tissues from Altay sheep (ALS, fat-rumped), Wadi sheep (WDS, long-tailed), Small-tailed Han sheep (STH, short-tailed), Chinese Merino sheep (CMS, long thin-tailed), and Tibetan sheep (TS, short thin-tailed) populations in summer.

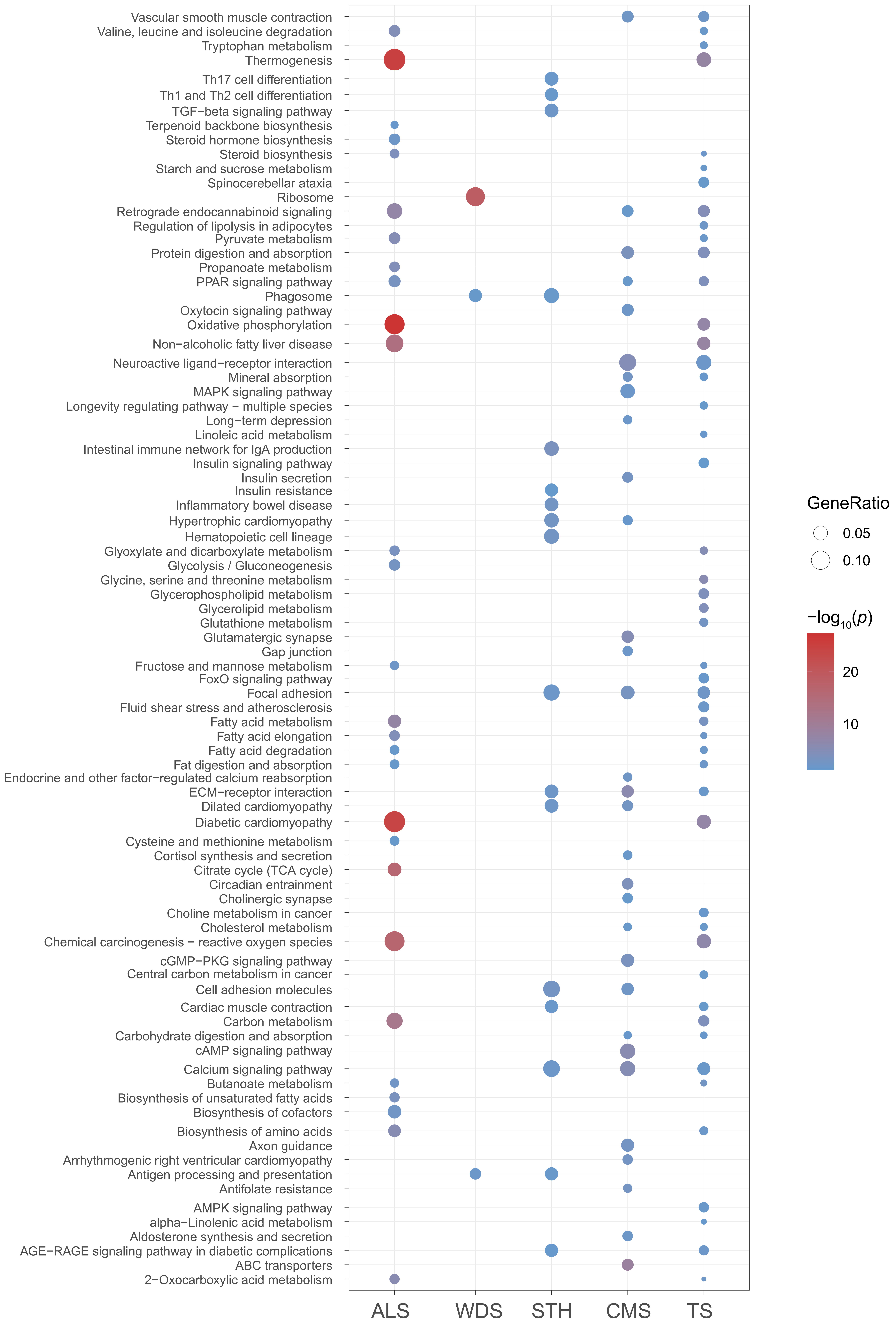

Supplementary Figure 4. Significantly (*P*adj. < 0.05) enriched Kyoto Encyclopedia of Genes and Genomes (KEGG) pathways for the differentially expressed genes (DEGs) upregulated in the adipose tissues from Altay sheep (ALS, fat-rumped), Wadi sheep (WDS, long-tailed), Small-tailed Han sheep (STH, short-tailed), Chinese Merino sheep (CMS, long thin-tailed), and Tibetan sheep (TS, short thin-tailed) populations in summer.

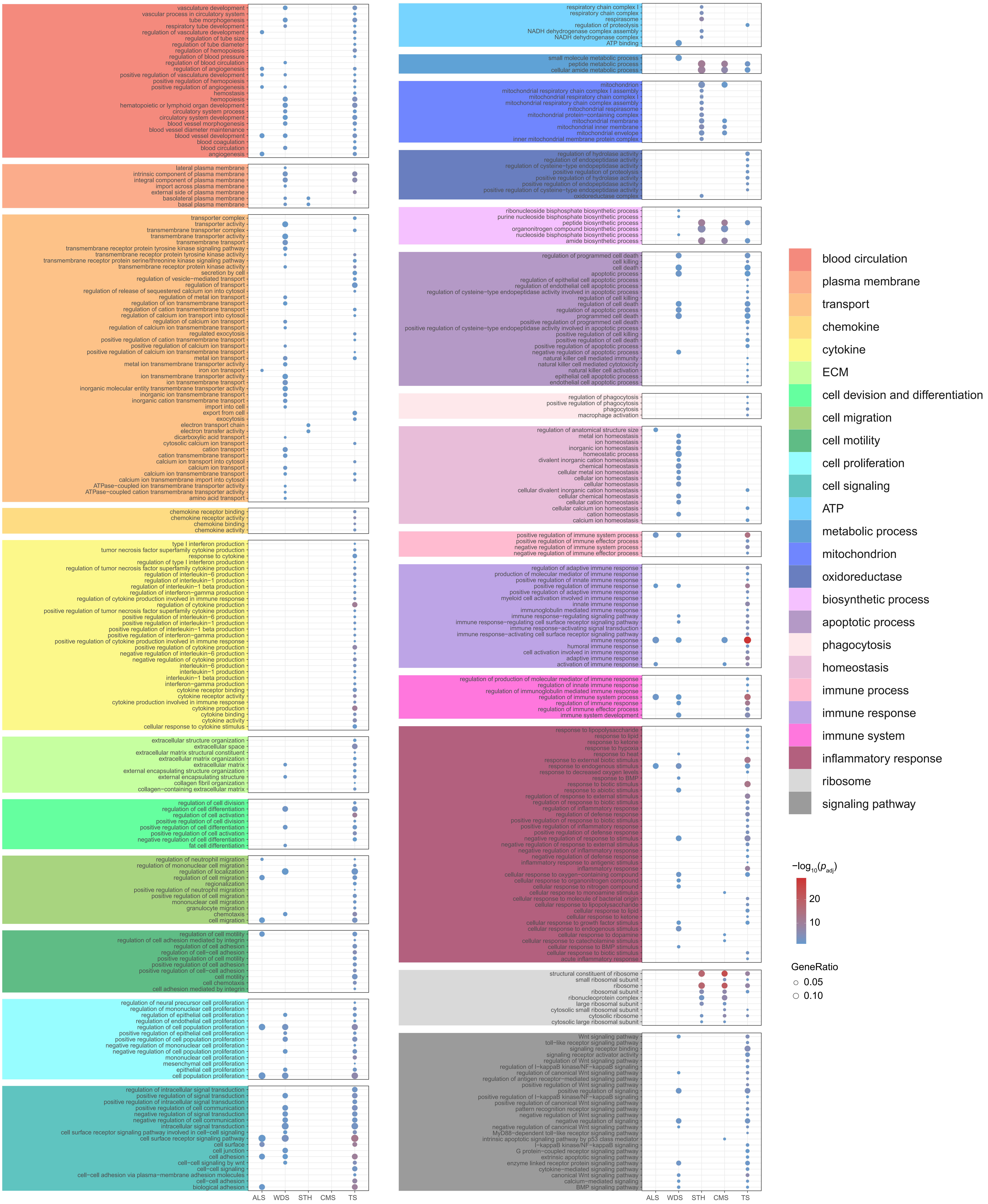

Supplementary Figure 5. Significantly (*P*adj. < 0.05) enriched gene ontology (GO) terms for the differentially expressed genes (DEGs) upregulated in the adipose tissues from Altay sheep (ALS, fat-rumped), Wadi sheep (WDS, long-tailed), Small-tailed Han sheep (STH, short-tailed), Chinese Merino sheep (CMS, long thin-tailed), and Tibetan sheep (TS, short thin-tailed) populations in winter.

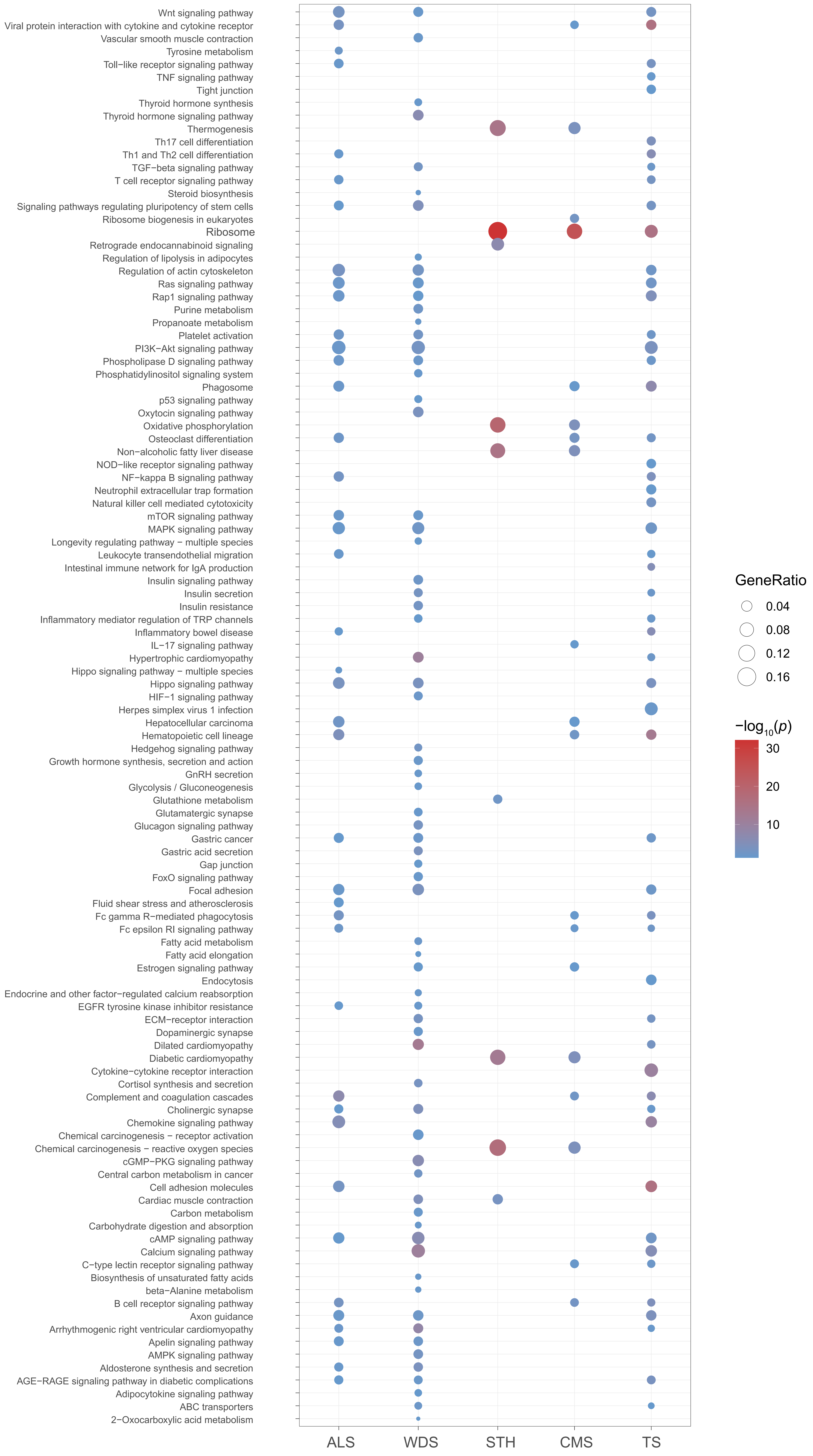

Supplementary Figure 6. Significantly (*P*adj. < 0.05) enriched Kyoto Encyclopedia of Genes and Genomes (KEGG) pathways for the differentially expressed genes (DEGs) upregulated in the adipose tissues from Altay sheep (ALS, fat-rumped), Wadi sheep (WDS, long-tailed), Small-tailed Han sheep (STH, short-tailed), Chinese Merino sheep (CMS, long thin-tailed), and Tibetan sheep (TS, short thin-tailed) populations in winter.

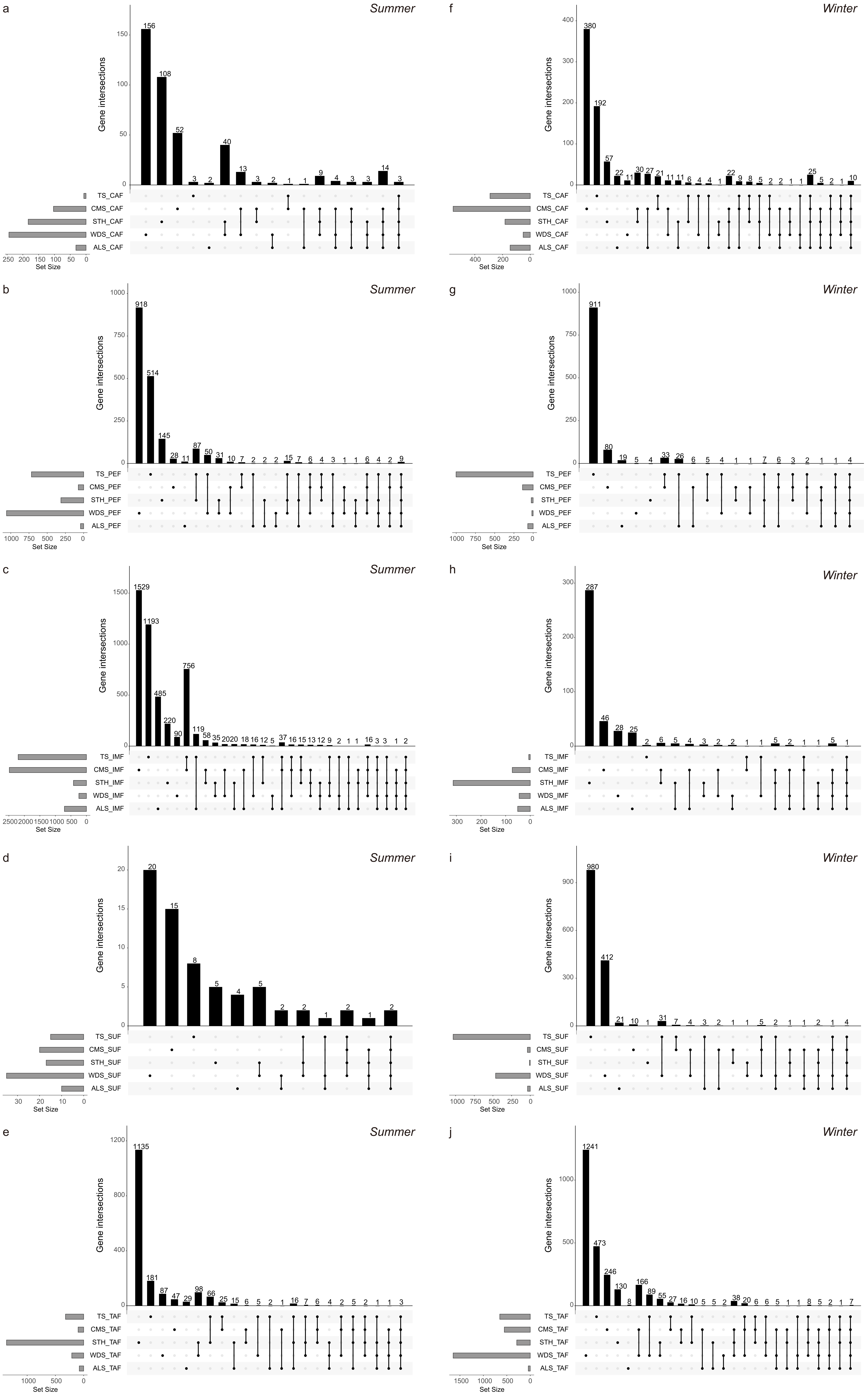

Supplementary Figure 7. **UpSet plot of depot-specific differentially expressed genes (DEGs) from RNA-Seq data from the five different adipose tissues**. **a**-**e**. Number of upregulated DEGs specific to the adipose tissues of caul fat (**a**), perirenal fat (**b**), intermuscular fat (**c**), subcutaneous fat (**d**), and tail fat (**e**) in the 5 populations in summer. **f**-j. Number of upregulated DEGs specific to the adipose tissues of caul fat (**f**), perirenal fat (**g**), intermuscular fat (**h**), subcutaneous fat (**i**), and tail fat (**j**) in the 5 populations in winter.

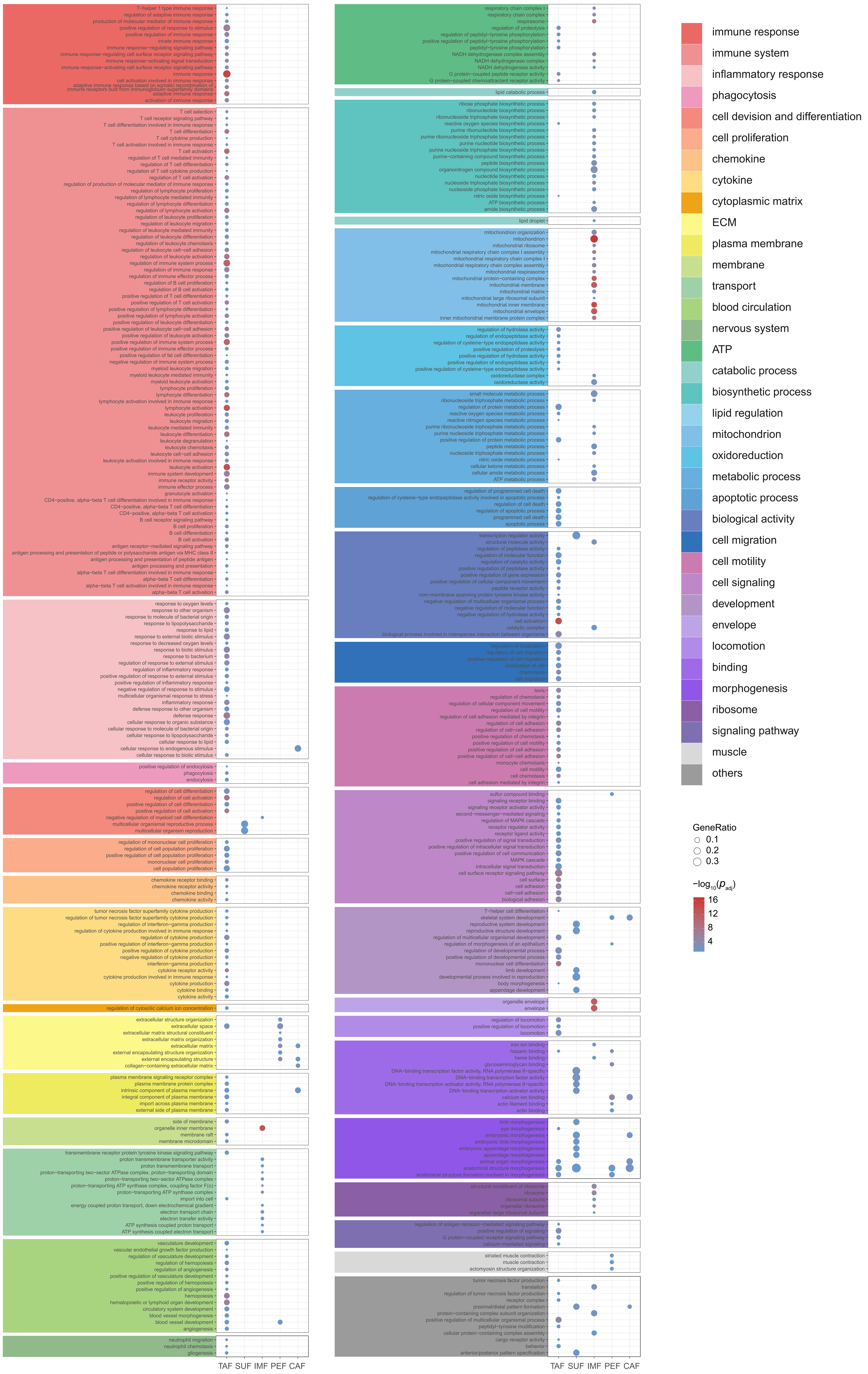

Supplementary Figure 8. Significantly (*P*adj. < 0.05) enriched gene ontology (GO) terms for the differentially expressed genes (DEGs) upregulated in the adipose tissues of tail fat (TAF), subcutaneous fat (SUF), intermuscular fat (IMF), perirenal fat (PEF), and caul fat (CAF) in summer.

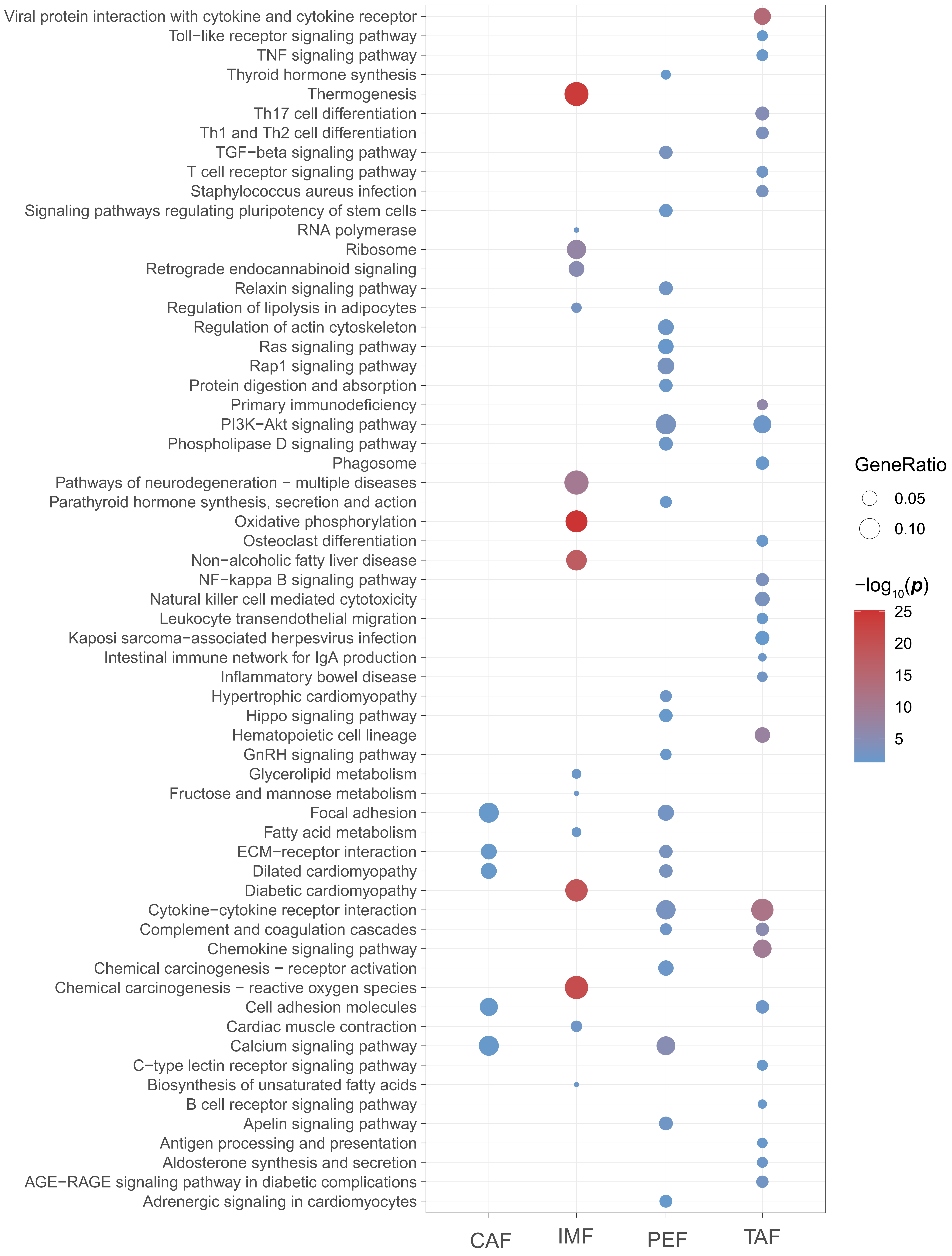

Supplementary Figure 9. Significantly (*P*adj. < 0.05) enriched Kyoto Encyclopedia of Genes and Genomes (KEGG) pathways for the differentially expressed genes (DEGs) upregulated in the adipose tissues of tail fat (TAF), subcutaneous fat (SUF), intermuscular fat (IMF), perirenal fat (PEF), and caul fat (CAF) in summer.

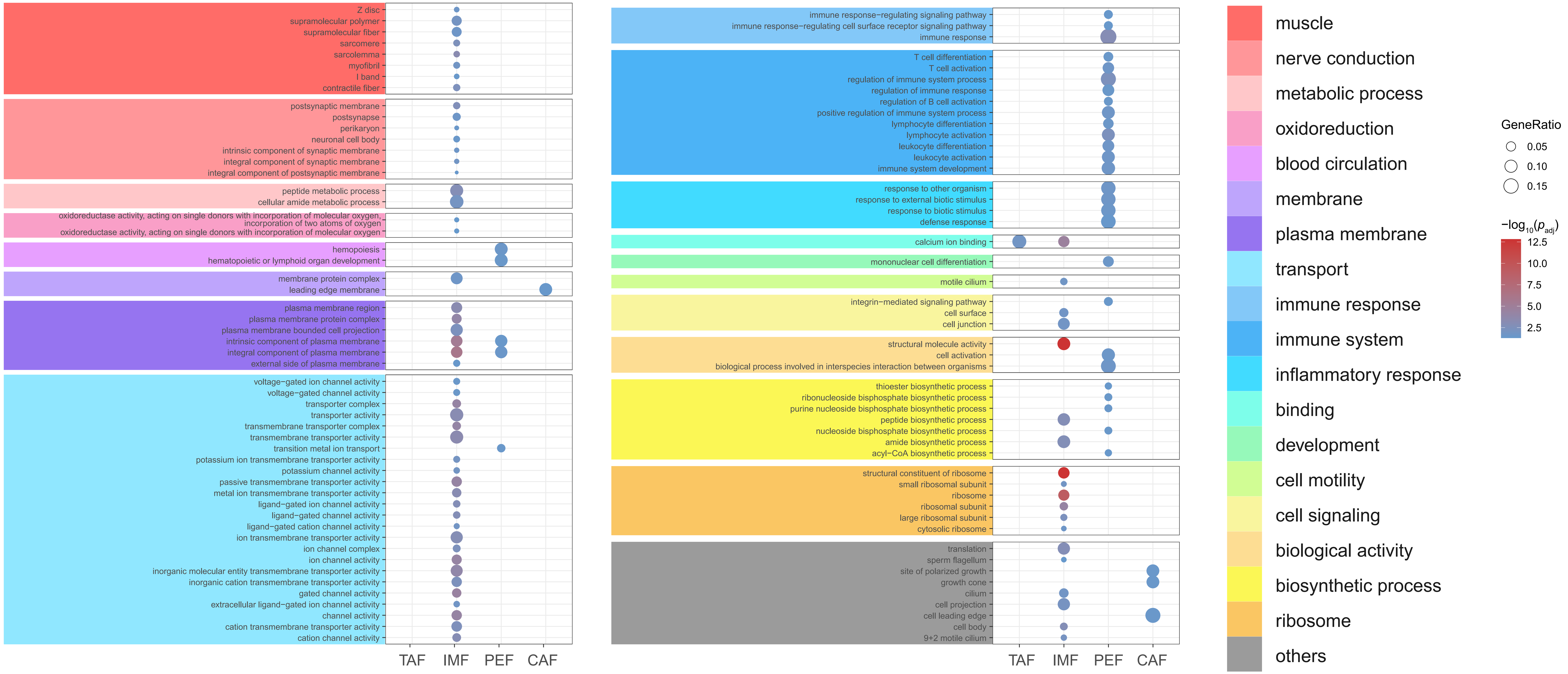

Supplementary Figure 10. Significantly (*P*adj. < 0.05) enriched gene ontology (GO) terms for the differentially expressed genes (DEGs) downregulated in the adipose tissues of tail fat (TAF), subcutaneous fat (SUF), intermuscular fat (IMF), perirenal fat (PEF), and caul fat (CAF) in summer

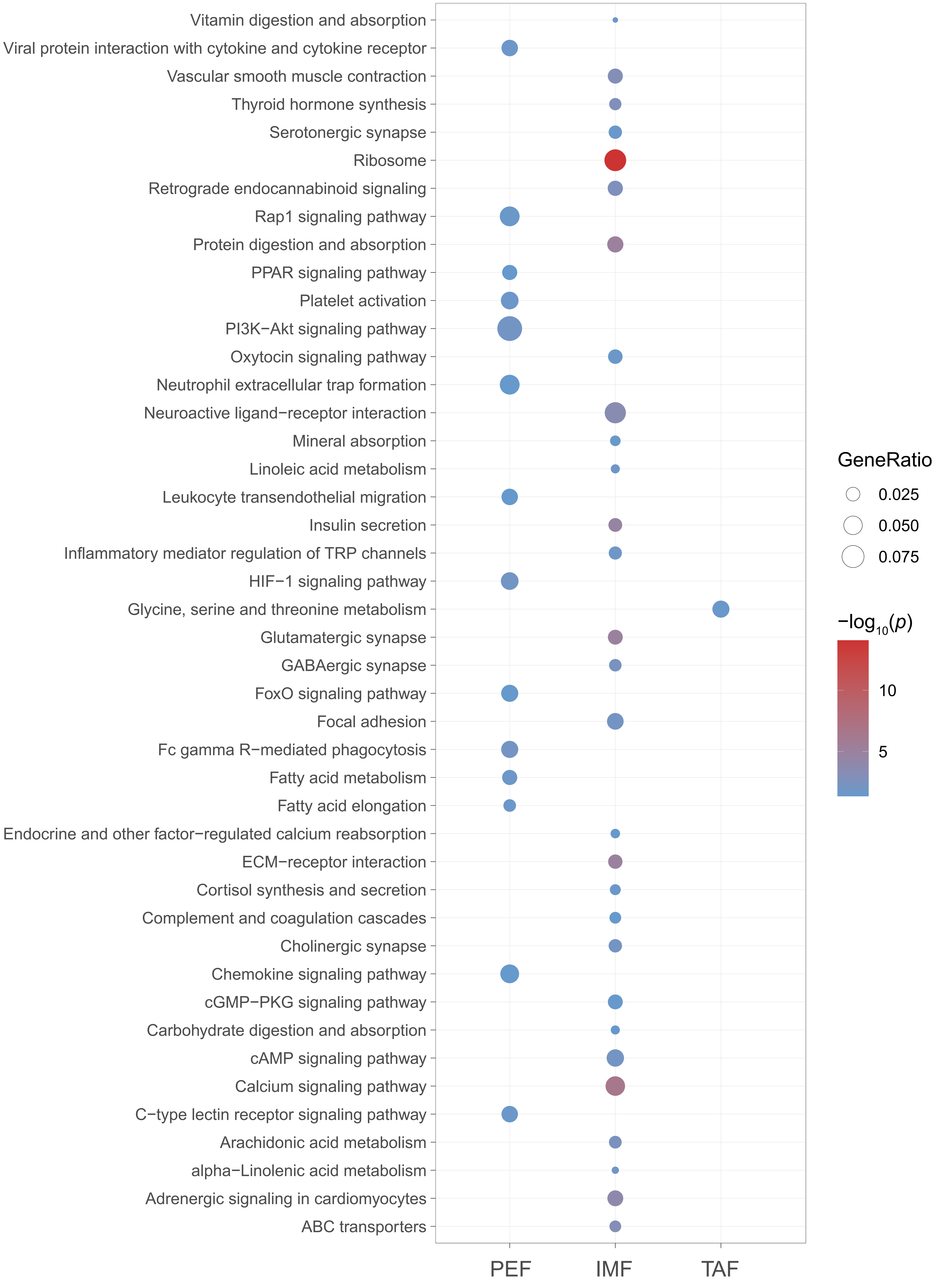

Supplementary Figure 11. Significantly (*P*adj. < 0.05) enriched Kyoto Encyclopedia of Genes and Genomes (KEGG) pathways for the differentially expressed genes (DEGs) downregulated in the adipose tissues of tail fat (TAF), subcutaneous fat (SUF), intermuscular fat (IMF), perirenal fat (PEF), and caul fat (CAF) in summer.

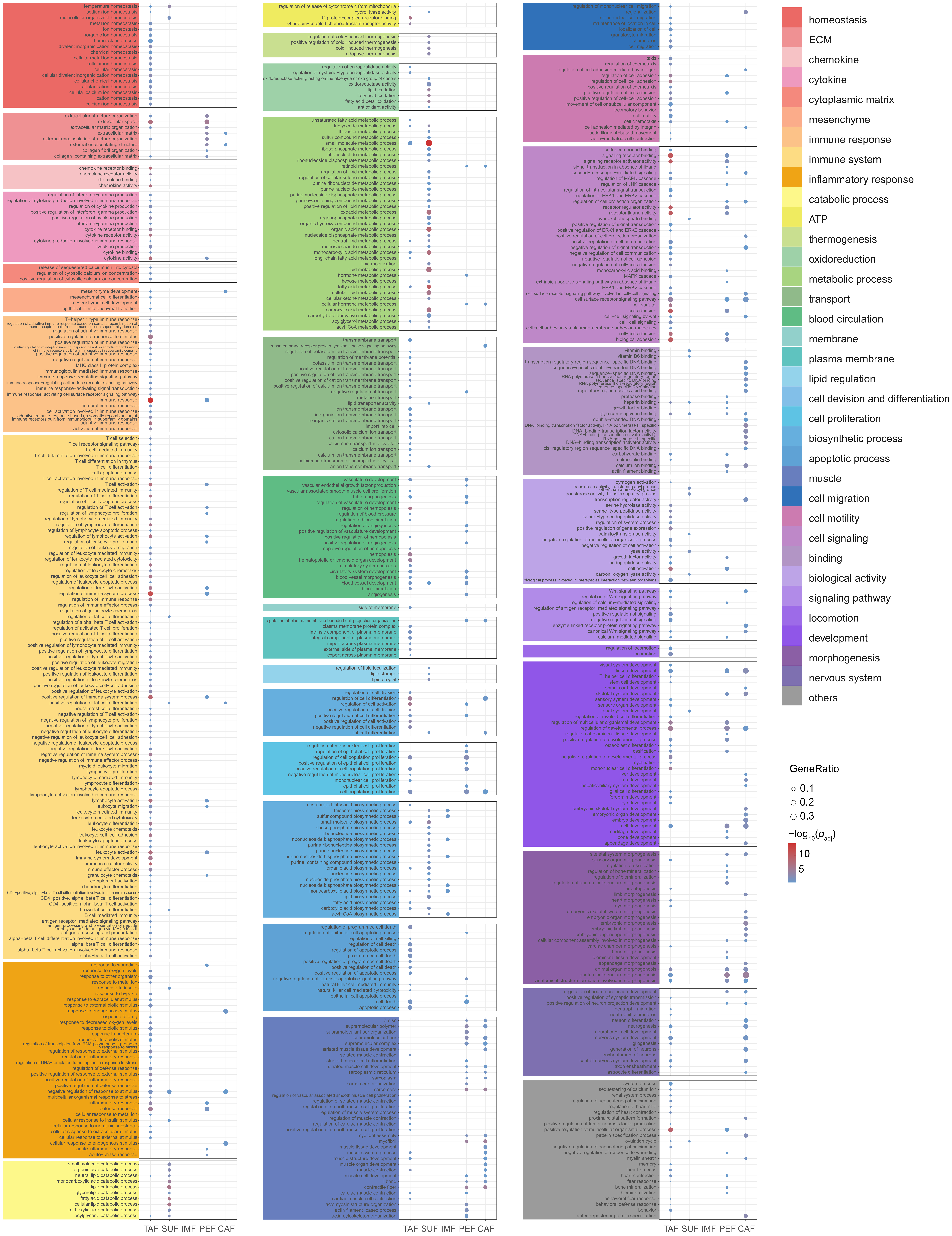

Supplementary Figure 12. Significantly (*P*adj. < 0.05) enriched gene ontology (GO) terms for the differentially expressed genes (DEGs) upregulated in the adipose tissues of tail fat (TAF), subcutaneous fat (SUF), intermuscular fat (IMF), perirenal fat (PEF), and caul fat (CAF) in winter.

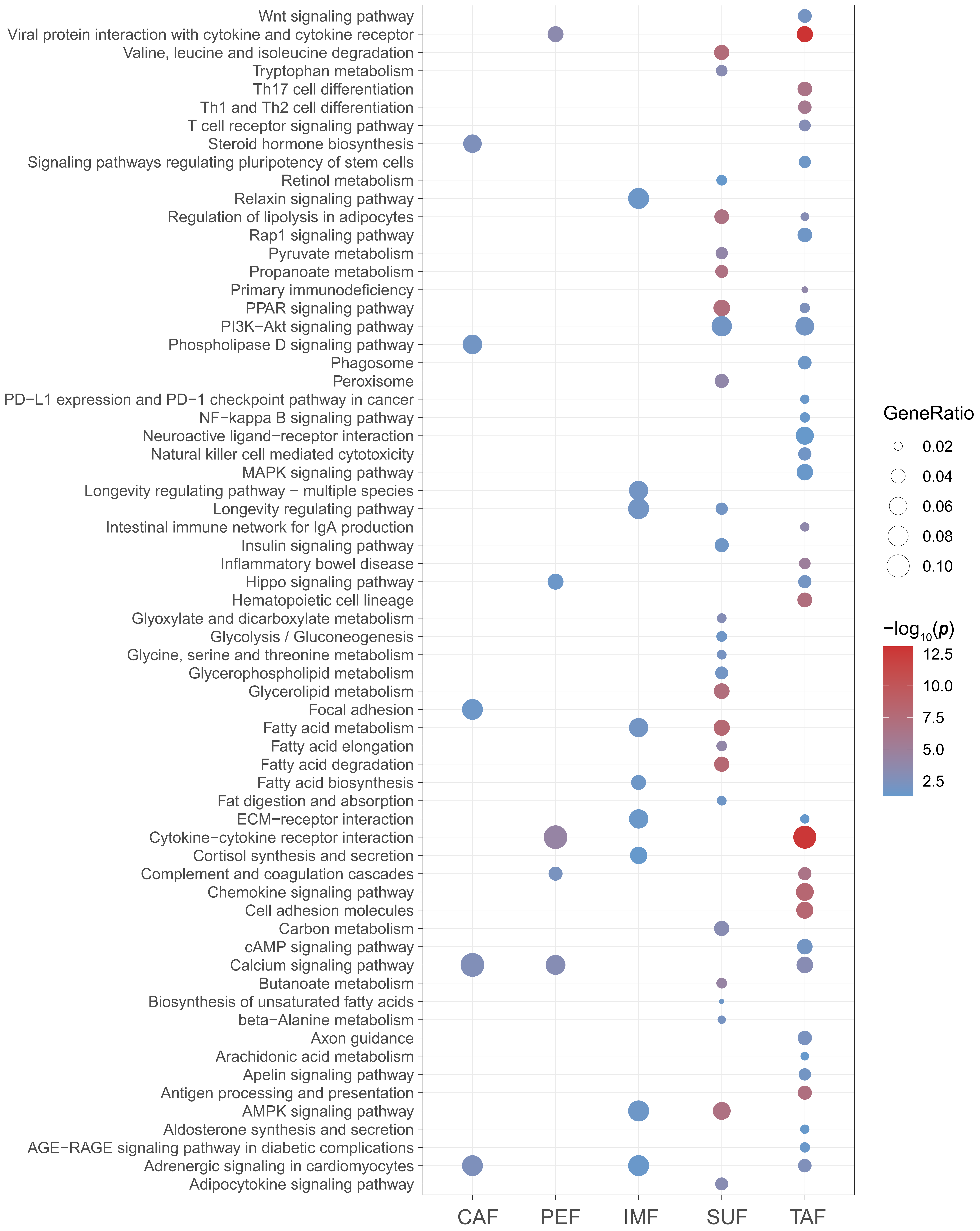

Supplementary Figure 13. Significantly (*P*adj. < 0.05) enriched Kyoto Encyclopedia of Genes and Genomes (KEGG) pathways for the differentially expressed genes (DEGs) upregulated in the adipose tissues of tail fat (TAF), subcutaneous fat (SUF), intermuscular fat (IMF), perirenal fat (PEF), and caul fat (CAF) in winter.

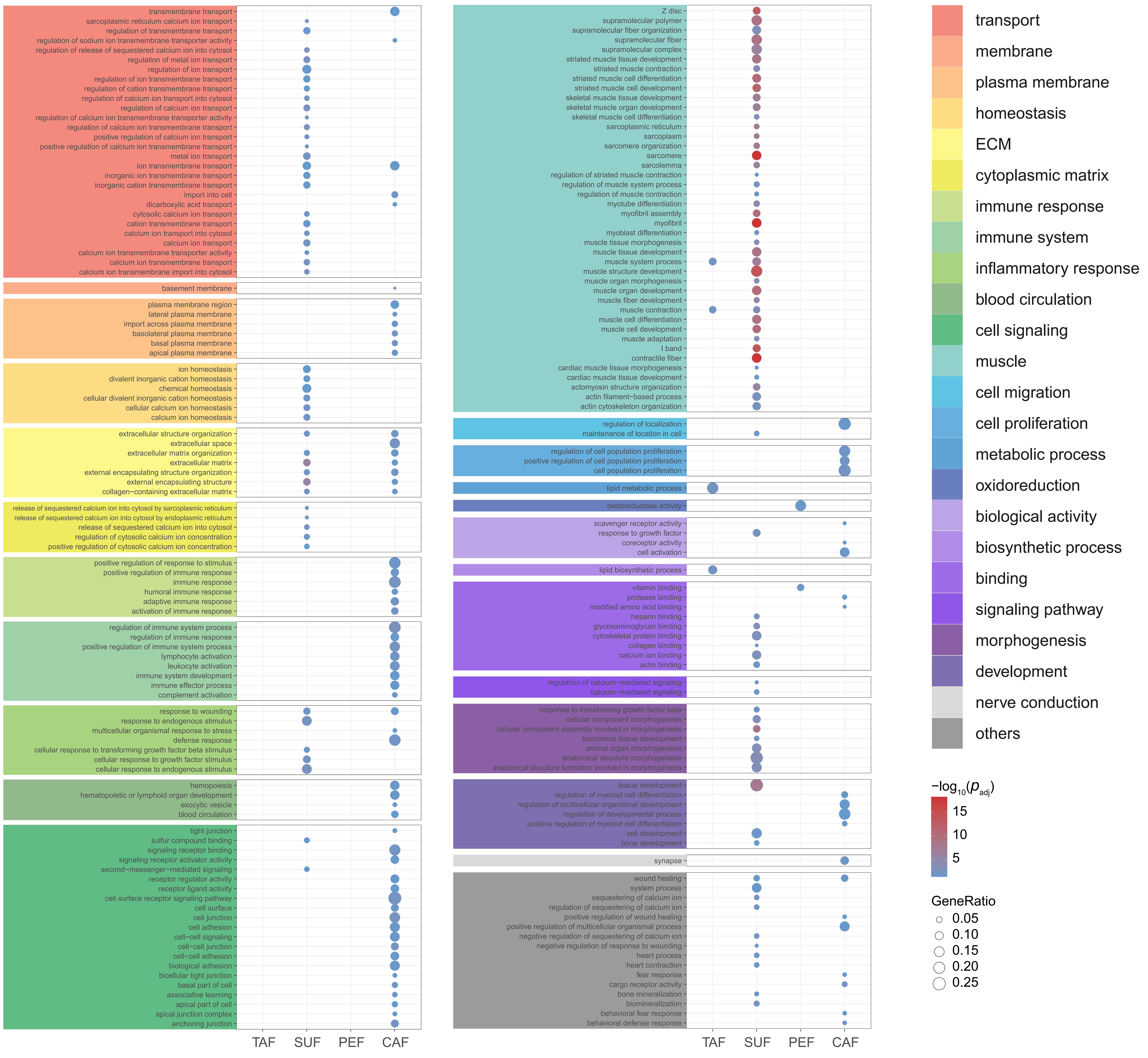

Supplementary Figure 14. Significantly (*P*adj. < 0.05) enriched gene ontology (GO) terms for the differentially expressed genes (DEGs) downregulated in the adipose tissues of tail fat (TAF), subcutaneous fat (SUF), intermuscular fat (IMF), perirenal fat (PEF), and caul fat (CAF) in winter.

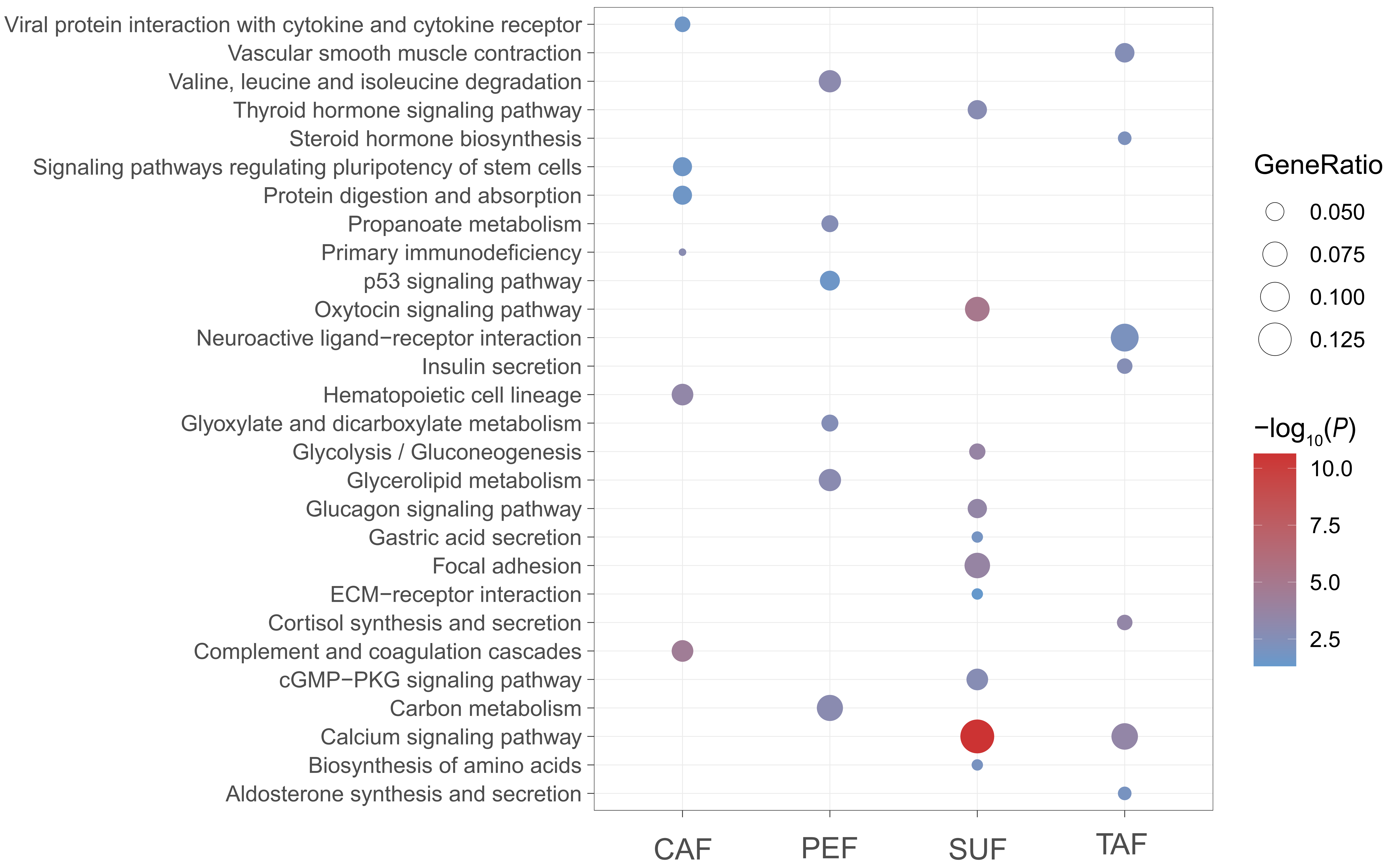

Supplementary Figure 15. Significantly (*P*adj. < 0.05) enriched Kyoto Encyclopedia of Genes and Genomes (KEGG) pathways for the differentially expressed genes (DEGs) downregulated in the adipose tissues of tail fat (TAF), subcutaneous fat (SUF), intermuscular fat (IMF), perirenal fat (PEF), and caul fat (CAF) in winter.

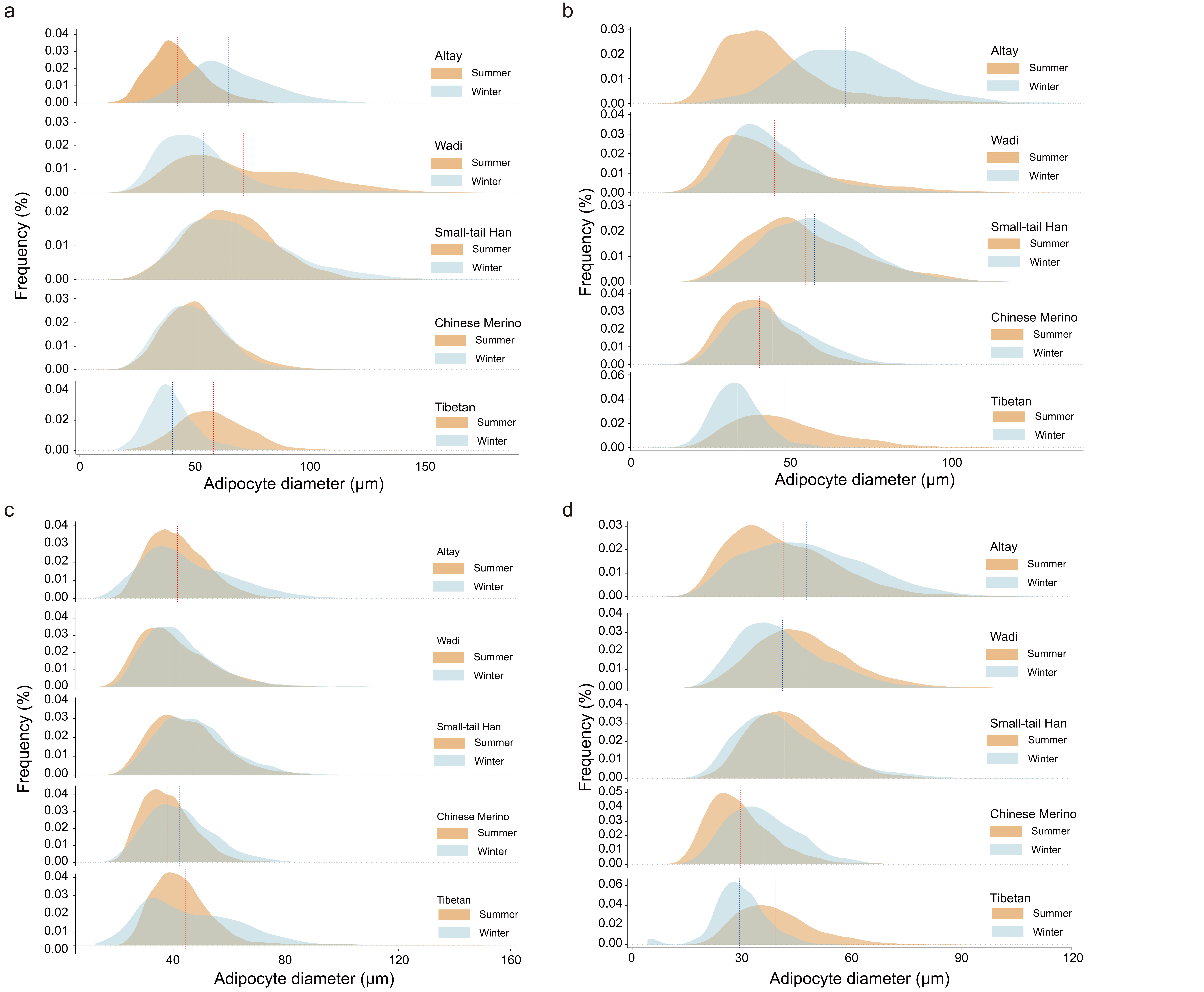

Supplementary Figure 16. **Variations in cell size**. The size distribution of adipocytes of caul fat (**a**), perirenal fat (**b**), intermuscular fat (**c**), and subcutaneous fat (**d**) from Altay sheep, Wadi sheep, Small-tailed Han sheep, Chinese Merino sheep, and Tibetan sheep in summer and winter. The red dashed lines represent the observed mean diameter value for adipocytes in summer, and the blue dashed lines represent the observed mean diameter value for adipocytes in winter.

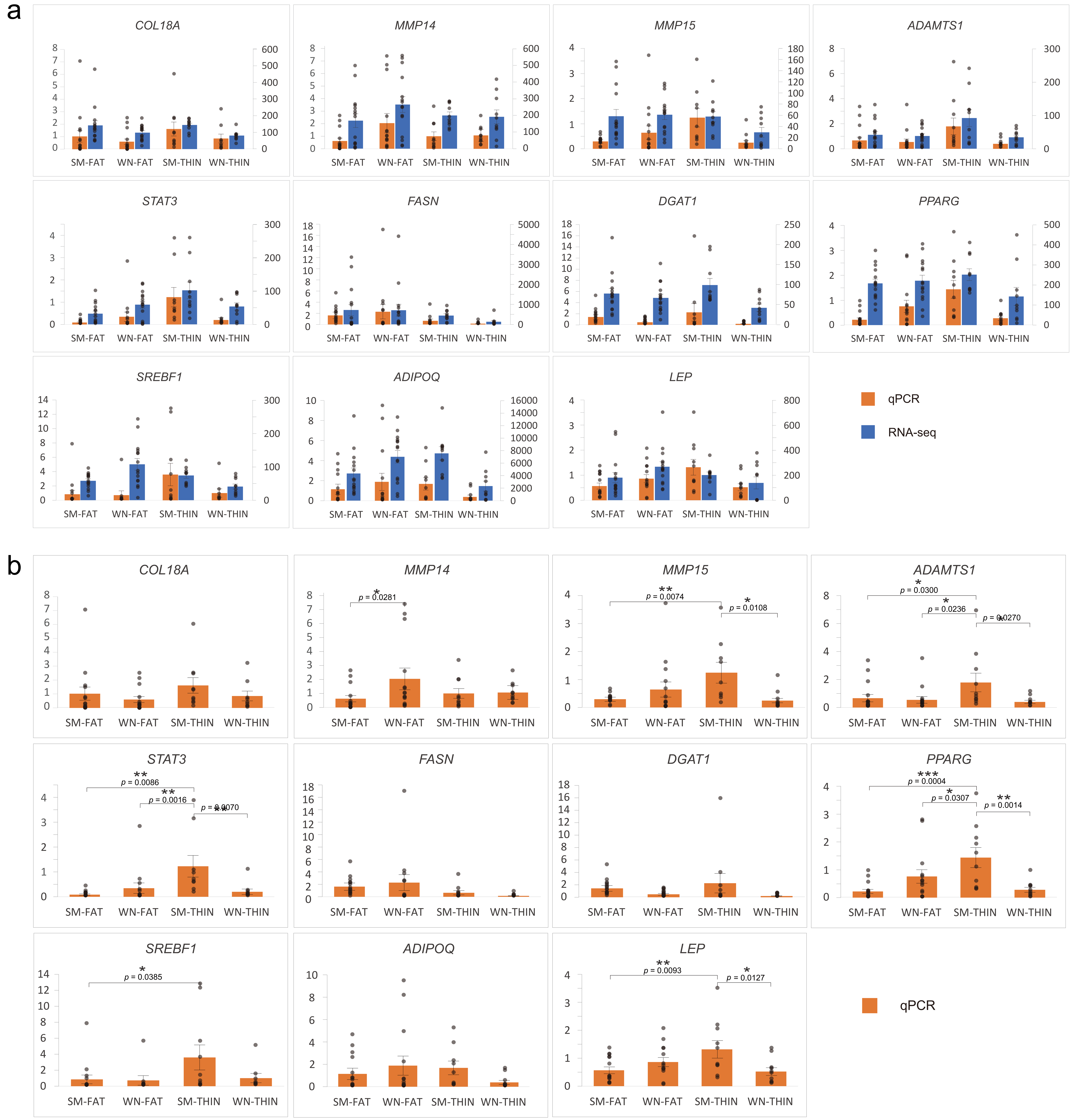

Figure S17.**RT-qPCR validation**. (**a**) RT-qPCR validation of RNA-sequencing (RNA-seq) data using 11 genes related to ECM remodeling, lipid droplet dynamic, and inflammation in summer fat tail adipose tissues (SM-FAT), winter fat tail adipose tissues (WN-FAT), summer thin-tail adipose tissues (SM-THIN), and winter thin-tail adipose tissues (WN-THIN). (**b**) RT-qPCR validation expression patterns among 4 groups of tail adipose tissues using 11 genes related to ECM remodeling, lipid droplet dynamic, and inflammation. The upper and boundary lines of the boxes represent the standard error of the mean.

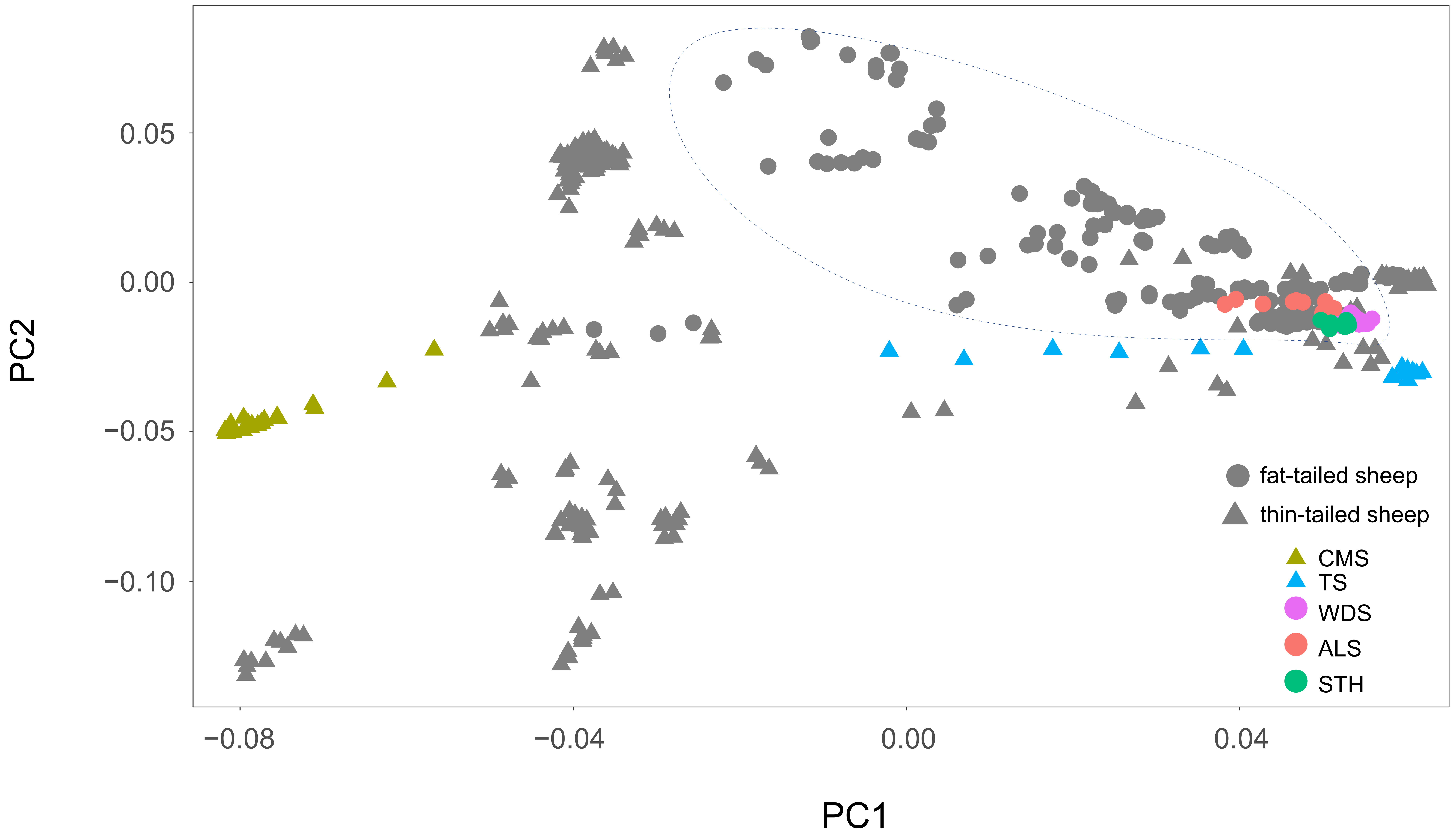

Supplementary Figure 18. Principal Component Analysis (PCA) plot showing the multivariate variation among 525 sheep used for genomic analyses.

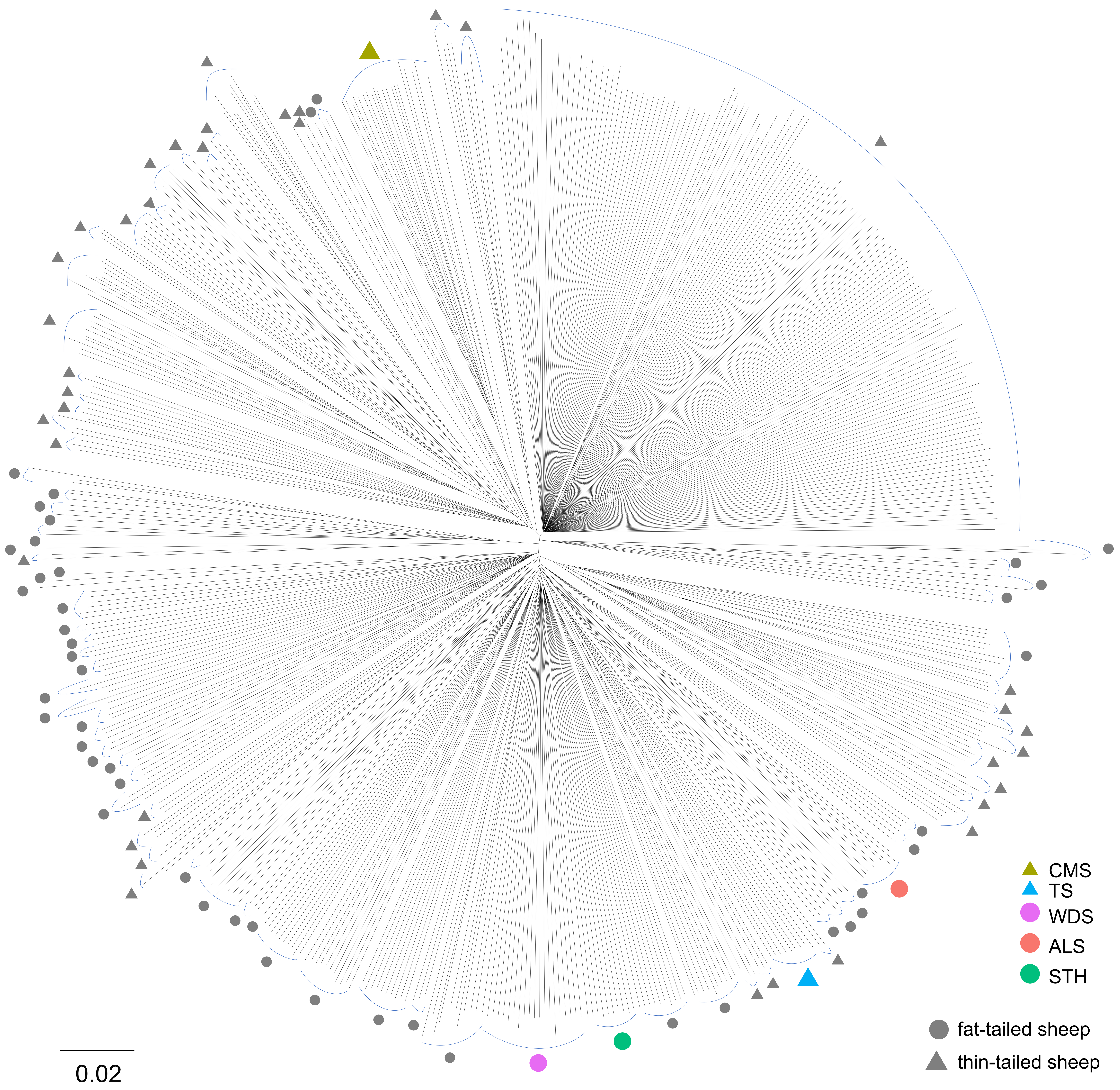

Supplementary Figure 19. Neighbor-joining (NJ) tree diagram showing the genetic relationship that was determined using genetic distances of 525 sheep with different tail types.

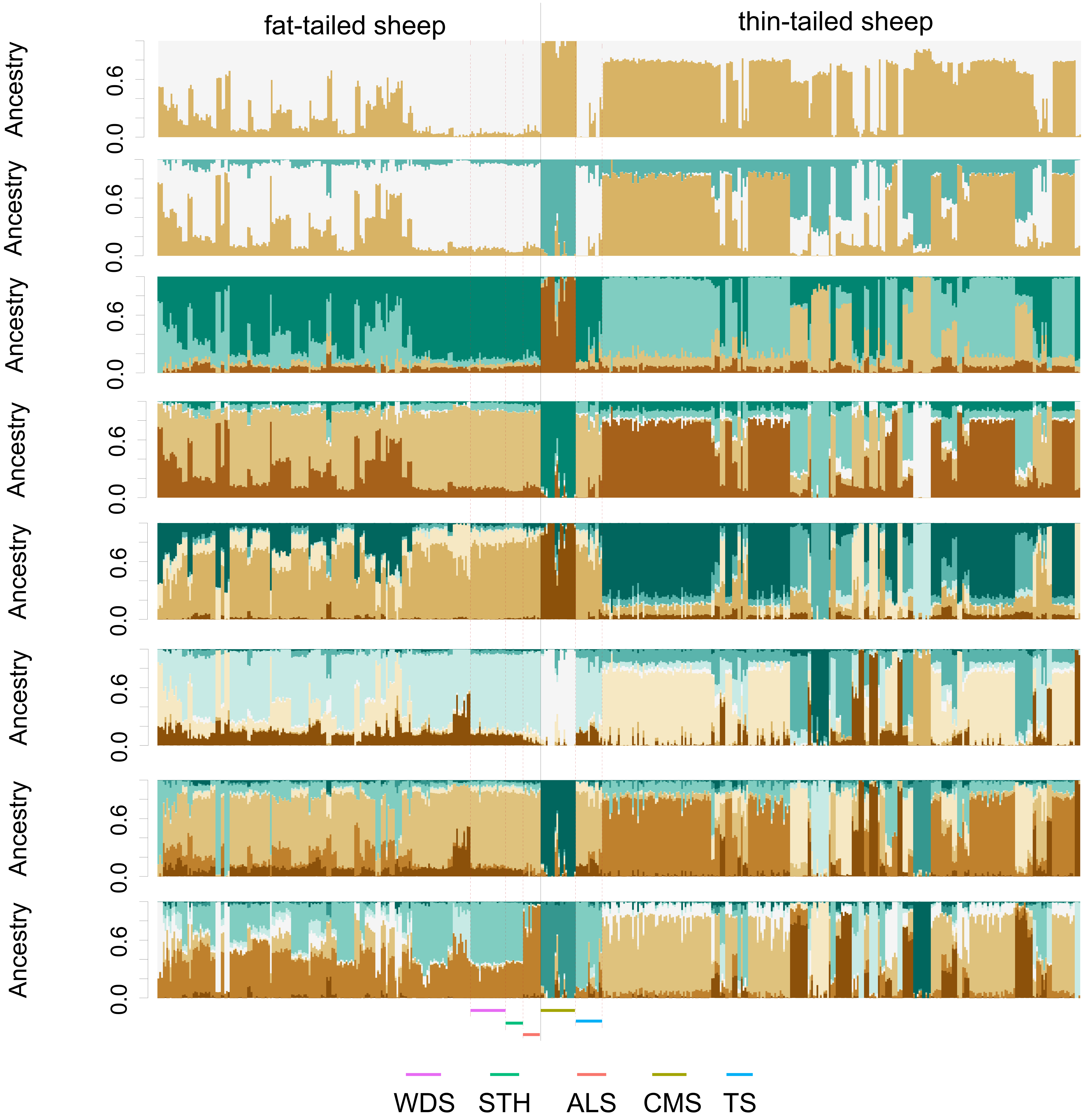

Supplementary Figure 20. Population structure of the 221 fat-tailed sheep and 304 thin-tailed sheep estimated by sNMF from K = 2 to K = 9.

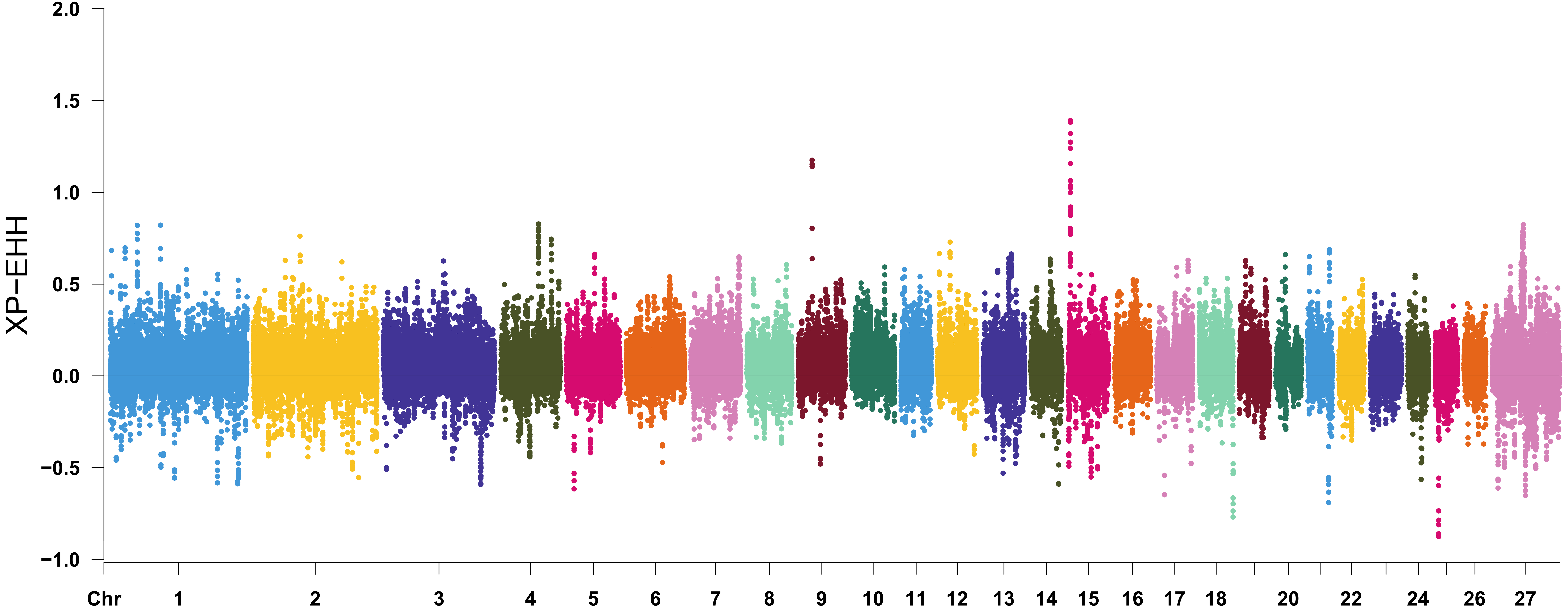

Supplementary Figure 21. Genome-wide selective signals between fat-tailed sheep and thin-tailed sheep by the cross-population extended haplotype homozygosity (XP-EHH) test. The horizontal red dashed line corresponds to the genome-wide significance threshold (top 5%: 0.4559).
