# Supplementary material for: Whole-body adipose tissue multi-omic analyses in sheep reveal molecular mechanisms underlying local adaptation to extreme environments
Source: Commun Biol. 2023 Feb 8;6:159. doi: 10.1038/s42003-023-04523-9 (PMC9908986; doi:10.1038/s42003-023-04523-9)
Supplement: Supplementary file 2 — Description of Additional Supplementary Files [file 42003_2023_4523_MOESM2_ESM.pdf]

Description of Additional Supplementary Data Files

File name: Supplementary Data 1

Description: Sampling information of the 250 adipose tissues in this study.

File name: Supplementary Data 2

Description: Summary statistics information of RNA-Seq data and alignment statistics for the 250 samples.

File name: Supplementary Data 3

Description: The source data for Figure 2a.

File name: Supplementary Data 4

Description: The source data for Figure 2b.

File name: Supplementary Data 5

Description: The source data for Figure 2c.

File name: Supplementary Data 6

Description: The source data for Supplementary Figure 3, 4.

File name: Supplementary Data 7

Description: The source data for Supplementary Figure 5,6.

File name: Supplementary Data 8

Description: A list of significantly enriched GO terms and KEGG pathways with genes significantly upregulated in summer in tail fats of fat-tailed and thin-tailed populations.

File name: Supplementary Data 9

Description: A list of significantly enriched GO terms and KEGG pathways with genes

significantly upregulated in winter in tail fats of fat-tailed and thin-tailed populations.

File name: Supplementary Data 10

Description: The source data for Figure 2d and Supplementary Figure 7.

File name: Supplementary Data 11

Description: The source data for Figure 2e.

File name: Supplementary Data 12

Description: The source data for Figure 2f.

File name: Supplementary Data 13

Description: The source data for Figure 2h.

File name: Supplementary Data 14

Description: The source data for Figure 2i.

File name: Supplementary Data 15

Description: The source data for Supplementary Figure 12.

File name: Supplementary Data 16

Description: The source data for Supplementary Figure 13.

File name: Supplementary Data 17

Description: The source data for Supplementary Figure 14.

File name: Supplementary Data 18

Description: The source data for Supplementary Figure 15.

File name: Supplementary Data 19

Description: The raw data source of the targeted lipidomics detected in the 250 samples for Figure 3a.

File name: Supplementary Data 20

Description: The raw data source of Lipidomic profiling of the 250 samples for Figure 3b, c.

File name: Supplementary Data 21

Description: The source data for Figure 3d.

File name: Supplementary Data 22

Description: The source data for Figure 3e.

File name: Supplementary Data 23

Description: The source data for Figure 3f.

File name: Supplementary Data 24

Description: The source data for Figure 3g

File name: Supplementary Data 25

Description: The source data for Figure 3h.

File name: Supplementary Data 26

Description: The source data for Figure 3i.

File name: Supplementary Data 27

Description: The source data for Figure 3k.

File name: Supplementary Data 28

Description: The source data for Figure 3l.

File name: Supplementary Data 29

Description: The source data for Figure 4a.

File name: Supplementary Data 30

Description: The source data for Figure 4b.

File name: Supplementary Data 31

Description: The source data for Figure 4c.

File name: Supplementary Data 32

Description: The source data for Figure 4c.

File name: Supplementary Data 33

Description: The source data for Figure 4h

File name: Supplementary Data 34

Description: The source data for Supplementary Figure 17a, b.

File name: Supplementary Data 35

Description: Summary information of whole-genome sequences in selective sweep tests.

File name: Supplementary Data 36

Description: A list of putative genomic regions under selection based on the top 5‰ values of global F_{ST}.

File name: Supplementary Data 37

Description: A list of putative genomic regions under selection based on the top 5‰ values of global ln(π ratio)/ln(2).

File name: Supplementary Data 38

Description: A list of putative genomic regions under selection based on the top 5‰ values of global XP-CLR.

File name: Supplementary Data 39

Description: A list of putative genomic regions under selection based on the top 5‰ values of global XP-EHH.

File name: Supplementary Data 40

Description: A list of overlapping regions under selection identified with the top 5‰ F_{ST} and top 5‰ ln(π ratio)/ln(2).

File name: Supplementary Data 41

Description: A list of overlapping regions under selection identified with the top 5‰ FST and top 5‰ XP-CLR.

File name: Supplementary Data 42

Description: A list of overlapping regions under selection identified with the top 5‰ XP-CLR and top 5‰ ln(π ratio)/ln(2).

File name: Supplementary Data 43

Description: A list of functional annotations of the overlapping genes under selection identified with at least two of the top 5‰ XP-CLR, top 5‰ ln(π ratio)/ln(2), and top 5‰ F_{ST}.

File name: Supplementary Data 44

Description: A list of overlapping regions under selection identified between the top 5‰ XP-EHH and top 5‰ XP-CLR, top 5‰ ln(π ratio)/ln(2), and top 5‰ F_{ST}.

File name: Supplementary Data 45

Description: A list of significant SNP variants (Z-test) under selective regions detected by at least two of the three methods based on the estimates of F_{ST}, π ratio, and XP-CLR.
