# Supplementary material for: Whole-body adipose tissue multi-omic analyses in sheep reveal molecular mechanisms underlying local adaptation to extreme environments
Source: Commun Biol. 2023 Feb 8;6:159. doi: 10.1038/s42003-023-04523-9 (PMC9908986; doi:10.1038/s42003-023-04523-9)
Supplement: Supplementary file 4 — Reporting Summary [file 42003_2023_4523_MOESM4_ESM.pdf]

nature portfolio

Corresponding author(s): Feng-Hua Lv

Last updated by author(s): Jan 13, 2023

Reporting Summary

Nature Portfolio wishes to improve the reproducibility of the work that we publish. This form provides structure for consistency and transparency in reporting. For further information on Nature Portfolio policies, see our Editorial Policies and the Editorial Policy Checklist.

Statistics

For all statistical analyses, confirm that the following items are present in the figure legend, table legend, main text, or Methods section.

n/a	Confirmed	
☐	☒	The exact sample size (*n*) for each experimental group/condition, given as a discrete number and unit of measurement
☐	☒	A statement on whether measurements were taken from distinct samples or whether the same sample was measured repeatedly
☒	☐	The statistical test(s) used AND whether they are one- or two-sided<br>*Only common tests should be described solely by name; describe more complex techniques in the Methods section.*
☒	☐	A description of all covariates tested
☐	☒	A description of any assumptions or corrections, such as tests of normality and adjustment for multiple comparisons
☐	☒	A full description of the statistical parameters including central tendency (e.g. means) or other basic estimates (e.g. regression coefficient) AND variation (e.g. standard deviation) or associated estimates of uncertainty (e.g. confidence intervals)
☒	☐	For null hypothesis testing, the test statistic (e.g. *F*, *t*, *r*) with confidence intervals, effect sizes, degrees of freedom and *P* value noted<br>*Give P values as exact values whenever suitable.*
☒	☐	For Bayesian analysis, information on the choice of priors and Markov chain Monte Carlo settings
☐	☒	For hierarchical and complex designs, identification of the appropriate level for tests and full reporting of outcomes
☒	☐	Estimates of effect sizes (e.g. Cohen's *d*, Pearson's *r*), indicating how they were calculated

Our web collection on statistics for biologists contains articles on many of the points above.

Software and code

Policy information about availability of computer code

Data collection	Whole-genome sequences of 525 domestic sheep were retrieved from six sources: the NextGen Consortium and five previous studies. Please see Table S34.
Data analysis	We used open source to analyses the data in this sutdy. All open source used in this study, such as Trimmomatic v0.36, BWA men v0.7.8, GATK v4.1.2.0, VCFtools v0.1.17, PLINK v1.90, EIGENSOFT v.6.0.1, SplitsTree v4.17.1, Figtree v1.4.4, DESeq2 v3.12, Cytoscape, ClusterProfiler,R v4.1.0, ComplexHeatmap v1.20.2, MetaboAnalystR 3.0, Kyoto Encyclopedia of Genes and Genomes Compound database (http://www.kegg.jp/kegg/compound/), KEGG pathway database (http://www.kegg.jp/kegg/pathway.html), XP-CLR v1.0, and Selscan 2.0.0, were freely accessed.

For manuscripts utilizing custom algorithms or software that are central to the research but not yet described in published literature, software must be made available to editors and reviewers. We strongly encourage code deposition in a community repository (e.g. GitHub). See the Nature Portfolio guidelines for submitting code & software for further information.

Data

Policy information about availability of data

All manuscripts must include a data availability statement. This statement should provide the following information, where applicable:

- Accession codes, unique identifiers, or web links for publicly available datasets
- A description of any restrictions on data availability
- For clinical datasets or third party data, please ensure that the statement adheres to our policy

The raw sequencing data have been deposited in the NCBI SRA under Bioproject numbers XXXXX. Whole-genome sequences of 525 domestic sheep were retrieved from six sources: the NextGen Consortium and five previous studies. Please see Table S34

Human research participants

Policy information about studies involving human research participants and Sex and Gender in Research.

Reporting on sex and gender	*Use the terms sex (biological attribute) and gender (shaped by social and cultural circumstances) carefully in order to avoid confusing both terms. Indicate if findings apply to only one sex or gender; describe whether sex and gender were considered in study design whether sex and/or gender was determined based on self-reporting or assigned and methods used. Provide in the source data disaggregated sex and gender data where this information has been collected, and consent has been obtained for sharing of individual-level data; provide overall numbers in this Reporting Summary. Please state if this information has not been collected. Report sex- and gender-based analyses where performed, justify reasons for lack of sex- and gender-based analysis.*
Population characteristics	*Describe the covariate-relevant population characteristics of the human research participants (e.g. age, genotypic information, past and current diagnosis and treatment categories). If you filled out the behavioural & social sciences study design questions and have nothing to add here, write "See above."*
Recruitment	*Describe how participants were recruited. Outline any potential self-selection bias or other biases that may be present and how these are likely to impact results.*
Ethics oversight	*Identify the organization(s) that approved the study protocol.*

Note that full information on the approval of the study protocol must also be provided in the manuscript.

Field-specific reporting

Please select the one below that is the best fit for your research. If you are not sure, read the appropriate sections before making your selection.

☒ Life sciences ☐ Behavioural & social sciences ☐ Ecological, evolutionary & environmental sciences

For a reference copy of the document with all sections, see nature.com/documents/nr-reporting-summary-flat.pdf

Life sciences study design

All studies must disclose on these points even when the disclosure is negative.

Sample size	Following the previous studies similar with this study, we chosen samples size. For transpcriptomic and lipidomic experiments, we collected 5 samples for each population. In the five populations, we collected adipose tissues from five different body fat depots (subcutaneous, tail, perirenal, intermuscular, and caul fat) for RNA-Seq and lipid profiling, consisting of 25 animals in summer (June – August) and 25 animals in winter (November – January).
Data exclusions	All data generated in this study were used to perform analyses.
Replication	The experiments, e.g. RT-qPCR, were replicated three times with similar results.
Randomization	No randomization were used in this study.
Blinding	No blinding were used in this study.

Reporting for specific materials, systems and methods

We require information from authors about some types of materials, experimental systems and methods used in many studies. Here, indicate whether each material, system or method listed is relevant to your study. If you are not sure if a list item applies to your research, read the appropriate section before selecting a response.

Materials & experimental systems

n/a	Involved in the study
☒	☐ Antibodies
☒	☐ Eukaryotic cell lines
☐	☐ Palaeontology and archaeology
☐	☒ Animals and other organisms
☒	☐ Clinical data
☒	☐ Dual use research of concern

Methods

n/a	Involved in the study
☒	☐ ChIP-seq
☒	☐ Flow cytometry
☒	☐ MRI-based neuroimaging

Palaeontology and Archaeology

Specimen provenance	*Provide provenance information for specimens and describe permits that were obtained for the work (including the name of the issuing authority, the date of issue, and any identifying information). Permits should encompass collection and, where applicable, export.*
Specimen deposition	*Indicate where the specimens have been deposited to permit free access by other researchers.*
Dating methods	*If new dates are provided, describe how they were obtained (e.g. collection, storage, sample pretreatment and measurement), where they were obtained (i.e. lab name), the calibration program and the protocol for quality assurance OR state that no new dates are provided.*

☐ Tick this box to confirm that the raw and calibrated dates are available in the paper or in Supplementary Information.

Ethics oversight	*Identify the organization(s) that approved or provided guidance on the study protocol, OR state that no ethical approval or guidance was required and explain why not.*

Note that full information on the approval of the study protocol must also be provided in the manuscript.

Animals and other research organisms

Policy information about studies involving animals; ARRIVE guidelines recommended for reporting animal research, and Sex and Gender in Research

Laboratory animals	50 ewes (2 – 3 years old) of Tibetan sheep, Chinese Merino, Small-tailed Han, Wadi, and Altay sheep, representing the typical short-thin-tailed, long-thin-tailed, short-fat-tailed, long-fat-tailed, and fat-rumped sheep populations, were selected respectively.
Wild animals	The study did not involved wild animals.
Reporting on sex	Sex was considered in study design. All samples used in this study were female.
Field-collected samples	The study did not involve field collected samples.
Ethics oversight	The animal study was reviewed and approved by Laboratory Animal Welfare and Animal Experimental Ethical Committee of China Agricultural University. Animal sampling was also approved by local authorities where the samples were taken.

Note that full information on the approval of the study protocol must also be provided in the manuscript.
